# RabA2b Overexpression Alters the Plasma-Membrane Proteome and Improves Drought Tolerance in Arabidopsis

**DOI:** 10.3389/fpls.2021.738694

**Published:** 2021-10-06

**Authors:** Vivek Ambastha, Ifat Matityahu, Dafna Tidhar, Yehoram Leshem

**Affiliations:** ^1^Department of Plant Sciences, MIGAL – Galilee Research Institute, Kiryat Shmona, Israel; ^2^Faculty of Sciences and Technology, Tel-Hai College, Upper Galilee, Israel

**Keywords:** RabA2b, small GTPase, vesicle trafficking, water stress, drought, osmotic stress, ABA, *Arabidopsis thaliana*

## Abstract

Rab proteins are small GTPases that are important in the regulation of vesicle trafficking. Through data mining, we identified RabA2b to be stress responsive, though little is known about the involvement of RabA in plant responses to abiotic stresses. Analysis of the RabA2b native promoter showed strong activity during osmotic stress, which required the stress hormone Abscisic acid (ABA) and was restricted to the vasculature. Sequence analysis of the promoter region identified predicted binding motifs for several ABA-responsive transcription factors. We cloned *RabA2b* and overexpressed it in Arabidopsis. The resulting transgenic plants were strikingly drought resistant. The reduced water loss observed in detached leaves of the transgenic plants could not be explained by stomatal aperture or density, which was similar in all the genotypes. Subcellular localization studies detected strong colocalization between RabA2b and the plasma membrane (PM) marker PIP2. Further studies of the PM showed, for the first time, a distinguished alteration in the PM proteome as a result of *RabA2b* overexpression. Proteomic analysis of isolated PM fractions showed enrichment of stress-coping proteins as well as cell wall/cuticle modifiers in the transgenic lines. Finally, the cuticle permeability of transgenic leaves was significantly reduced compared to the wild type, suggesting that it plays a role in its drought resistant properties. Overall, these data provide new insights into the roles and modes of action of RabA2b during water stresses, and indicate that increased RabA2b mediated PM trafficking can affect the PM proteome and increase drought tolerance.

## Introduction

*Ras-related proteins in brain* (Rab) proteins consist of a large family of small GTPases (20–25 KDa) with more than 60 Rab members identified in the human genome. The different Rab types play important roles in the regulation of all vesicle trafficking steps: from vesicle budding at the donor membrane, through vesicle transport, to vesicle docking and fusion with its target membrane (Stenmark, 2009; Hutagalung and Novick, 2011; Minamino and Ueda, 2019). In mammalians, the Rab11 sub-family is associated with the recycling endosome (RE) and the secretion/recycling pathways (Grant and Donaldson, 2009; Stenmark, 2009), and is typically represented by 1–3 members in genomes of animals or yeast (Zhang et al., 2007; Woollard and Moore, 2008). Intriguingly, in plant genomes, the RabA sub-family, which is related to mammalian Rab11, is highly diversified: in Arabidopsis, among the 57 identified Rab genes, 26 are members of the RabA sub-family (Rutherford and Moore, 2002; Woollard and Moore, 2008). Similar diversification of the RabA sub-family has been reported in other plant species such as rice (Zhang et al., 2007), cotton (Li and Guo, 2017), and poplar (Zhang et al., 2018).

In Arabidopsis, Rutherford and Moore further recognized six RabA subclasses, named RabA1–RabA6, which were not functionally characterized (Rutherford and Moore, 2002). Nielsen et al. (2008) reviewed several RabA studies in plants and indicated that some RabA members play a role in secretion and/or cell wall recycling processes. This suggestion was further supported by Lunn et al. (2013) who found that in several RabA mutants the profile of major cell wall components differed from the wild type (wt) profile. Therefore, these authors hypothesized that Golgi originating vesicles deliver specific types of cargos to the cell exterior and may be regulated by specific sub types of RabA proteins. Indeed, several intracellular localization studies reported that several Arabidopsis RabA members operate along the *trans*-Golgi network (TGN) – plasma membrane (PM) pathway, during cytokinesis and tip growth in the root and elongating pollen tubes (Chow et al., 2008; Szumlanski and Nielsen, 2009; Asaoka et al., 2013a; Nielsen, 2020).

Over the last decade it has been well established that the vesicular trafficking machinery plays an important role in the mediation of plant responses to a range of abiotic stresses (Leshem et al., 2006, 2007; Baral et al., 2015; Garcia de la Garma et al., 2015; Wang et al., 2020; Tripathy et al., 2021). Nevertheless, very little is known about the roles RabA proteins play in these responses. So far, only members of the RabA1 sub-family have been reported to be associated with abiotic stress responses in Arabidopsis, when the RabA1 quadruple mutant (*raba1a/raba1b/raba1c/raba1d*) was reported to exhibit a salt overly sensitive phenotype (Asaoka et al., 2013a). Yet, the precise mode of action these RabA1 members play during the cellular response to salinity and other abiotic stresses, remain unknown (Asaoka et al., 2013b). Recently, overexpression of *OsRab11*, which is the rice ortholog of *AtRabA1d* in Arabidopsis, was reported to improve the plant’s salt tolerance during mild/non-lethal salt treatment (100 mM NaCl) (Chen and Heo, 2018). However, the mode of action of how this rice ortholog affects the Arabidopsis native vesicular trafficking system is unknown. Interestingly, a RabA member was reported to be involved in the trafficking of the FLAGELLIN SENSING2 receptor to the plasma membrane (Choi et al., 2013). Moreover, overexpression of *RabA4c* in Arabidopsis enhanced the plant’s resistance to powdery mildew, due to enhanced callose deposition during early infection events (Ellinger et al., 2014). Therefore, RabA proteins can play role in biotic interactions as well (Nathalie Leborgne-Castel and Bouhidel, 2014; Tripathy et al., 2021).

Considering RabA suggested roles in PM trafficking, we postulated that more RabA members are involved in membrane trafficking to the PM during stress and membrane repair/recycling processes which are essential for stress tolerance (Lee et al., 2006; Wang et al., 2011a,b). In this study we examined a RabA2 member *-RabA2b*, whose transcription was identified via the Bio-Analytic microarray Resource (BAR), to be upregulated during several abiotic stresses. We analyzed the RabA2b promoter activity, and characterized RabA2b localization, functionality, and its effect on PM proteomics. Our findings offer novel insights into the roles and modes of action of RabA2b during water stresses.

## Materials and Methods

### Plant Materials and Growth Conditions

*Arabidopsis thaliana* wt plants of Col-0 and Ler-0 backgrounds were used in this study. Homozygous ABA insensitive (ABI) mutants *abi 1-1* (Ler-0) and *abi 2-1* (Ler-0) were obtained from the Arabidopsis Biological Resource Centre (ABRC, CS22, and CS23, respectively^1^), *abi 4-1* (Col-0) was a gift from Dr. Gad Miller. The T-DNA knockout mutant *raba2b-1* (GABI_638G09) was obtained from the Nottingham Arabidopsis Stock Centre (NASC^2^) and genotyped following GABI-KAT recommendations^3^ (see primers list in Supplementary Table 1). Seed sterilization and growth conditions were performed as described previously (Ambastha et al., 2020).

### Phylogenetic Analysis of RabA Sub-Family

For phylogenetic analysis, the protein sequence of the Rab A2 family was downloaded from NCBI^4^. Sequence alignment and phylogenetic analysis was performed using the default parameters in Clastal Omega (Sievers et al., 2011).

### Molecular Cloning and Plant Transformation

1.38 Kbp sequence of *AtRabA2b* gene and 1 Kbp of the promoter region (+1 to −1,000 Kbp) was amplified (Primer list – Supplementary Table 1), cloned in *pENTR_gene:RabA2b* and *pENTR_Pro:RabA2b* and verified by sequencing. pENTR_gene:*RabA2b* was cloned into gateway vector pK7WGF2.0 using Gateway^*TM*^ LR Clonase^*TM*^ enzyme mix (Invitrogen-11791019) to obtain *Pro35S:GFP-RabA2b*. Similarly, pENTR: promoter was cloned into pKGWFS7 and *ProRabA2b-GUS* reporter transcriptional fusion construct was designed (Karimi et al., 2002). Wt-plants (Col-0 and Ler-0) were floral dipped with Agrobacterium [*Agrobacterium tumefaciens* s. LBA4404 (Zhang et al., 2006)] followed by T0 seed selections on 1/2 MS Kanamycin (50 μg/ml). In total 10 and 14 independent transgenic lines of *Pro35S:GFP-RabA2b* and *ProRabA2b-GUS* were screened to obtain T3 homozygous plants. Similarly, the *abi1-1*, *abi2-1*, and *abi4-1* mutants were transformed with pRabA2b:GUS reporter construct. Transformants were selected on Kanamycin and T3 homozygous lines were isolated. The T3 RabA2b over expressing (OE) plants were further transformed as described above, with the specific plasma membrane marker Aquaporin *Pro35S:PIP2A-mCherry* plasmid (Nelson et al., 2007), which was obtained from ABRC (see Text Footnote 1, CD3-1008), following selections on 10 μg/ml Basta (Sigma 45520) to obtain T3 double transgene *Pro35S:GFP-RabA2b/Pro35S:PIP2-mCherry* plants. The details of vectors used are provided in Supplementary Table 2.

### Subcellular Colocalization of RabA2b and Microscopy

Five days old T3 double transgenic seedlings were further analyzed by confocal microscope LSM700 (Carl Zeiss, Germany) with Argon laser source fitted with Plan-Apochromat 20×/0.8M27 objective lenses. GFP and FM4-64 and mCherry were excited at 488 and 555 nm, respectively. Colocalization analysis was done using Zen 2.3 SP1 FP1 (black) software (Carl Zeiss, Germany) under the default setting. Image analysis was performed using Zen 2.5 (Blue edition) (Carl Zeiss, Germany) and ImageJ^5^.

### Drought Stress Treatment

Drought experiments were conducted as described earlier (Leshem et al., 2010; Wang et al., 2018). In brief, seeds of wild type-Col-0 (wt) and *RabA2b* overexpressing lines were sown in soil pots containing an equal amount of soil and placed together in watering treys which were irrigated equally for 5 weeks, after which gradual water stress was devised by withholding watering for several days (12 up to 17 days). Stress recovery was performed by resuming watering when sever wilting was observed in both wt-Col-0 and RabA2b OE lines and the recovery was monitored for 10 days. Four independent experiments were performed using five RabA2b OE lines to obtain data for statistical analyses.

### Leaf Water Loss Assays and Stomatal Aperture Measurements

In order to measure leaf water loss, five rosette leaves from five well irrigated individual 8-week-old plants were detached from the tested genotypes, and their initial fresh weight was measured. The leaves were then incubated in an open area on a working bench for up to 315 min. Leaf fresh weight was measured at an interval of every 15 min for the first 165 min, then at 30 min for the next 150 min. The water loss from detached leaves is expressed as the percentage change in the initial fresh weight as described by Leshem et al. (2010).

Stomatal apertures were determined in images of paradermal sections of the abaxial epidermis was captured using Motic AE2000 inverted microscope under 40× lenses. Leaves from a well-irrigated WT and RabA2b over-expiring plant were detached and placed in an open area on a working bench for 120 min for dehydrated samples. For the control sample, the measurement was performed just after detaching the leaves. The aperture size between the two guard-cells was measured using ImageJ software, according to Leshem et al. (2010).

### GUS Histochemical Assay

Twelve days old 1/2 MS grown Arabidopsis plants expressing *Pro:RabA2b-GUS* were supplemented with either 10 μM ABA (Sigma- A1049), 400mM Sucrose or 400 mM Mannitol for 24 h in liquid 1/2 MS followed by the GUS histochemical assay as described by Ambastha and Leshem (2020). The Image under lower magnification was captured by Stereo Microscope SZ61 (Olympus, Japan) equipped with a 5MP GXCAN-H5 camera (Stansfield, United Kingdom) while ultrastructure analysis was performed at 20× using inverted microscope AE2000 (Motic, Hong Kong) equipped with a Moticam3+ camera. GUS quantification was performed on ImageJ software (see Text Footnote 5) following the method described earlier (Ambastha and Leshem, 2020).

### Plasma-Membrane Enrichment, Protein Isolation, and Immunoblotting

Plasma membrane enriched fraction was isolated following protocol described by Santoni (2007) and protein was isolated in extraction buffer (50 mM NaHPO4, pH 7.0, 10 mM b-mercaptoethanol, 10 mM Na2EDTA, pH 8.0, 0.1% Sarcosyl, 0.1% Triton X-100). Total Proteins from wt Col-0, OE6.4, and OE11.4, as well as their plasma membrane enriched fraction (20 μg), were resolved on 12% (w/v) SDS-PAGE, transferred on to PVDF membrane and blocked with 4% (w/v) BSA at RT for 1 h. The membrane was labeled with an antibody against PIP2A (Agrisera- AS09491) or GFP (Abcam-183734) for overnight at 4°C. After washing in TBST-buffer (Tris-buffered saline, 0.1% Tween 20), the membrane was incubated in rabbit anti-goat HRP conjugate antibody (Abcam- ab6722) in TBS for 2–3 h at RT and washed again with TBST-buffer. Signal was detected using ECL solution.

### Mass Spectrometry, and Data Analysis

Acetone precipitated plasma membrane enriched protein was resuspended in Protein Solubilization buffer (9 M Urea and 100 mM ammonium bicarbonate), reduced with 3 mM DDT (60°C for 30 min) and finally alkylated with 10 mM IAA. This reduced and alkylated protein were digested over night at 37°C with trypsin (Promega-PR-V5280) in digestion buffer (1.5 M Urea and 25 mM ammonium bicarbonate). This tryptic digest was desalted using C18 Zip-tip and resuspended in 0.1% Formic acid. The desalted peptides in solvent A (0.1% formic acid in water) were injected in Q Exactive HF mass spectrometer (LC-MS/MS -Thermo) fitted with a capillary HPLC (easy nLC 1,000, Thermo) using homemade capillary column (20 cm, 75 micron ID) packed with Reprosil C18-Aqua (Dr. Maisch GmbH, Germany). A linear gradient of 5–28% for 105 min of solvent B (95% acetonitrile with 0.1% formic acid) was applied to resolve the peptides mixture followed by 15 min gradient of 28–95% and 15 min at 95% acetonitrile with 0.1% formic acid in water at flow rates of 0.15 μL/minute. Mass spectrometry was performed in a positive mode using repetitively full MS scan followed by high collision induces dissociation (HCD, at 35 normalized collision energy) of the 20 most dominant ions (>1 charges) selected from the first MS scan. The Data obtained were analyzed on MaxQuant software 1.5.2.8.^6^ using the Andromeda search engine, searching against the *Arabidopsis thaliana* proteome from the Uniprot database with mass tolerance of 20 ppm for the precursor masses and 20 ppm for the fragment ions. Peptide- and protein-level false discovery rates (FDRs) were filtered to 1% using the target-decoy strategy. Protein table was filtered to eliminate the identifications from the reverse database, common contaminants and single peptide identifications. The data was quantified by label free analysis using the same software, based on extracted ion currents (XICs) of peptides enabling quantitation from each LC/MS run for each peptide identified in any of experiments. Statistical analysis of the identification and quantization results was done using Perseus 1.6.7.0 software. The mass spectrometry proteomics data have been deposited to the ProteomeXchange Consortium via the PRIDE partner repository – http://www.ebi.ac.uk/pride (Perez-Riverol et al., 2019). Data are available via ProteomeXchange with identifier PXD028140.

### Gene Ontology Enrichment Analysis

Compare to the wild type, the significantly up-regulated proteins in the overexpressing lines (OE 6.4 and OE 6.11) were subjected to GO analysis using the “AgriGO v 2.0” web-based tool for gene ontology analysis (Du et al., 2010). Singular Enrichment Analysis (SEA) was applied to identify the biological processes or the molecular functions that are substantially enriched by the identified membrane proteins. “*A. thaliana* TAIR 10” was used as a reference for supported species while “Arabidopsis Genome locus (TAIR 10)” was used as a suggested background during SEA.

In addition, we checked the presence of TMH (Transmembrane helix) using the TMHMM Server (v. 2.0) web tool http://www.cbs.dtu.dk/services/TMHMM/. The network visualization of GO terms was performed using the ShinyGO tool http://bioinformatics.sdstate.edu/go/ (Ge et al., 2020).

### Database Mining

To search the ABA responsive nature of genes corresponding to differentially- abundant membrane proteins (DAMP), the hormone series data set of electronic northern browser^7^ was using default settings (Toufighi et al., 2005; Winter et al., 2007). After obtaining the electronic northern data for ABA treatment, genes with fourfold upregulation in expression were labeled as ABA responsive genes.

### Toluidine Blue Test

Leaf permeability assay using Toluidine blue was performed with slight modification as described by Tanaka et al. (2004). The rosette and cauline leaves of 4 weeks old plant was submerged in TB (Sigma-T3260) solution (0.05% W/V). After 2h, the TB solution was removed and the leaves were washed in running water to remove superficially attached TB solution.

## Results

### Genomic Features and Transcriptional Patterns of the RabA2 Sub-Family

**FIGURE 1:**
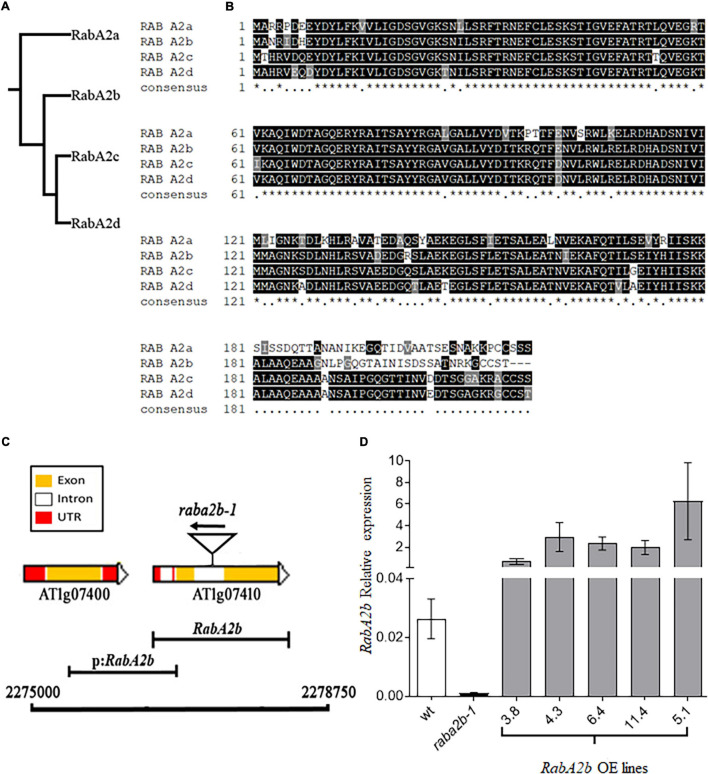
Molecular features *AtRabA2b*. **(A)** Phylogram of the RabA2 family using a multiple sequence alignment program (Clustal Omega) that uses seeded guide trees and HMM (Hidden Markov model) profile-profile techniques to generate alignments and phylogenetic tree. **(B)** Multiple sequence alignment, produced by ClustalW, aligns different RabA2 homologs according to their similarities in amino acid sequence. The amino acid shaded in Black, highlights the conserved residue, gray highlights the non-conserved amino acid but similar in properties and non-shaded residues are non-conserved. **(C)** Physical map of the *RabA2b* gene showing 3.75 kb region on Chromosome 1, between coordinates 2,27,500–2,24,500 comprising *RabA2b* gene (AT1g07410) and its promoter (*p:RabA2b*). The positions of the T-DNA (GABI_638G09) insertion is shown by the triangle. Color coding is for yellow, white and red is for – exon, intron and UTR region, respectively. The 1 kb putative promoter region cloned for tissue specific transcriptional fusion (GUS expression) in this study is represented by black capped line labeled as *p:RabA2b.* **(D)** Expression Analysis of *RabA2b* in over-expressing (OE) plants. Real-time PCR analysis of wild-type (WT), T-DNA insertion mutant *rabA2b1-1* (GABI_638G09) and RabA2b transgenic Arabidopsis plants, showing the expression in different T3 homozygous transgenic lines (line 3.8, 4.3, 6.4, and 11.4) with negligible expression in rabA2b mutant. Data are mean ± SE from three biological replicates each with three technical replicates. The “*” (asterisk) indicates positions which have a single, fully conserved amino acid residue.

Genomic analysis of the RabA family in Arabidopsis identified four members in the RabA2 sub-family and indicated that *RabA2b* (At1g07410) is distinct from *RabA2c* (At3g46830) and *RabA2d* (At5g59150) (Figure 1A). At the protein level, although high similarity exists between all members of the RabA2 sub-family, their C-terminus is significantly different (Figure 1B). Since the C-terminus of Rab proteins is generally known to be essential for binding with their specific target membranes (Chavrier et al., 1991; Li et al., 2014), this variation possibly provides each of the RabA2 members with unique roles and targets. To acquire transcriptional information of the RabA2 sub-family that was not available at 2002 (Rutherford and Moore, 2002), we mined the Bio-Analytic Resource - BAR (see Text Footnote 7) (Toufighi et al., 2005) and found that *RabA2b* displayed unique expression patterns. Unlike the other RabA2 members that were highly expressed throughout most of the stages of the plant life cycle, *RabA2b* remained mostly silent during these stages, except of high expression in the pollen (Supplementary Figure 1A). Nevertheless, *RabA2b* was highly upregulated by several abiotic stresses such as heat, drought and salt, while the rest of the RabA2 members generally responded marginally to these stresses (Supplementary Figure 1B).

### *RabA2b* Promoter Activity During Osmotic Stress

**FIGURE 2:**
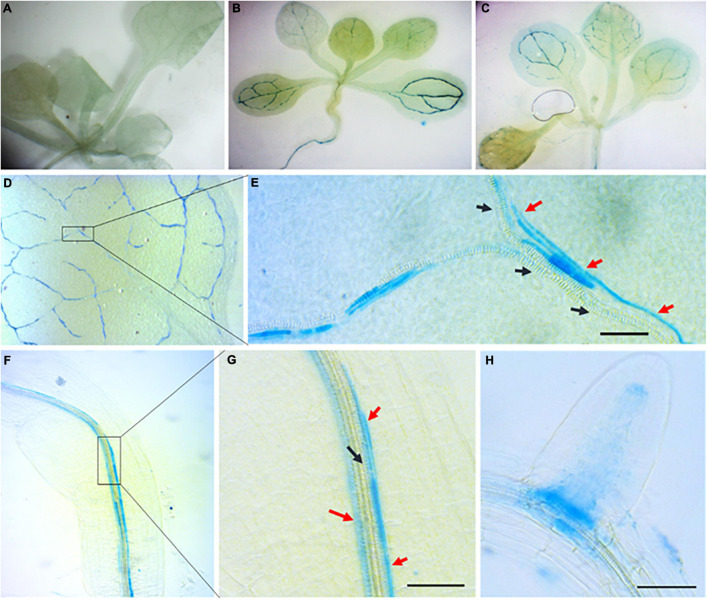
Promoter activity of *RabA2b* during water stresses and ABA treatment. GUS histochemical staining of transgenic Arabidopsis plants expressing GUS gene driven by *AtRabA2b* putative promoter in Col-0 background 12-h after osmotic stress (400 mM sucrose) or 10 μM ABA. The experiment was performed on 2 weeks old seedlings of five independent T3 homozygous lines. The representative images show plants under. **(A)** Control condition having no GUS staining, **(B)** sucrose induced osmotic stress with GUS expressing in leaves veins, and **(C)** 10 μM ABA supplemented with 1/2 MS. In **(D)** we observe a leaf showing GUS activity in the vasculatures, while **(E)** is an enlarged image of a leaf showing unstained proto-xylem and GUS staining in conducting elements running parallel to the xylem, suspected as the phloem. GUS expression in the root is observed in **(F)**, in **(G)**, a higher magnification of the boxed area in **(F)**, and in **(H)**, in young root. The Black and Red arrow in **(E,G)** indicates proto-xylem and suspected phloem elements, respectively. Scale bar = 50 μm. Similar results were obtained with 400 mM mannitol.

**FIGURE 3:**
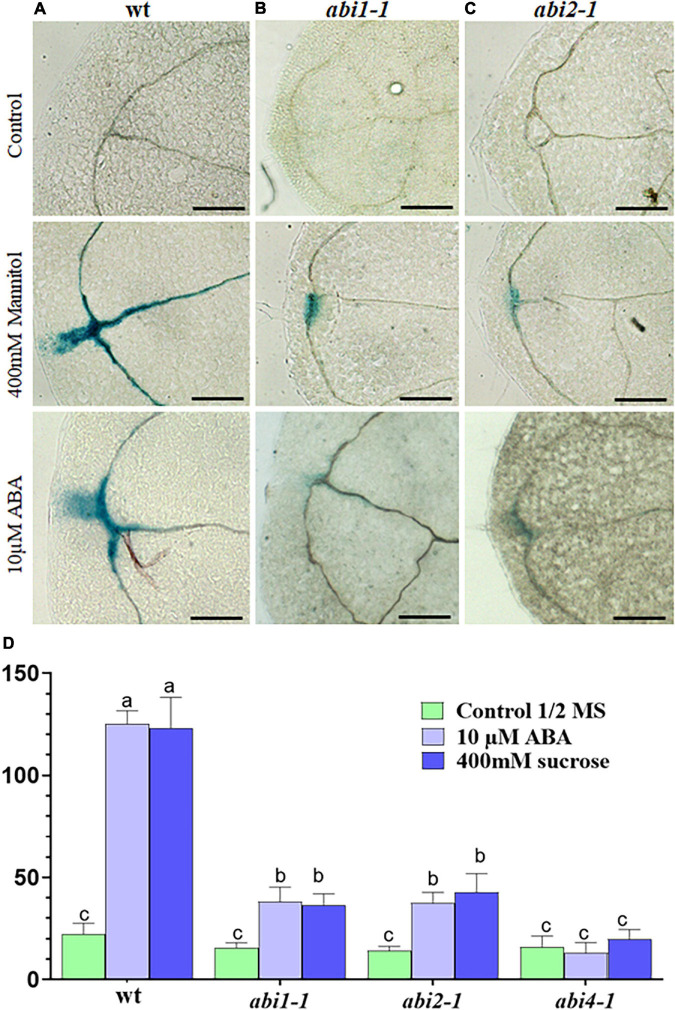
*RabA2b*promoter activity in *abi* mutants during water stress. **(A–C)** GUS enzymatic activity visualization and **(D)** histochemical staining quantification of transgenic Arabidopsis plant expressing GUS gene driven by *AtRabA2b* promoter in wt, *abi1.1* and *abi2.1* plants, 12-h after osmotic stress (400 mM sucrose) or 10 μM ABA. The experiment was performed three times, on three independent T3 homozygous lines. Images were captured to quantify GUS staining using ImageJ software as mentioned in Section “Materials and Methods.” The error bar in **(D)** indicates the standard error (SE) and significant differences between MS control, 400 mM sucrose and 10 μM ABA treated conditions were assessed with 2-way ANOVA multiple comparisons (*P* < 0.05). The letters (a, b, c) indicate the significant difference at *p* ≤ 0.05 Scale bar = 200 μm.

To further study *RabA2b* expression patterns during abiotic stresses we cloned the 1 KB genomic region upstream to its start codon, which is assumed to include the native promoter or part of it (Figure 1C). We then fused it to the GUS reporter gene and transformed it to Arabidopsis. GUS promoter analysis was performed in T3 homozygous plants. While during standard growth conditions GUS activity was not observed (Figure 2A), strong GUS activity was detected during osmotic stresses (mannitol and sucrose) (Figure 2B) or in response to ABA treatment of non-stressed plants (Figure 2C). The GUS signal was detected in both the shoot and the root tissues (Figures 2B–H). Surprisingly, the GUS signal was not spread evenly in all the tissues as we initially expected, but was rather restricted mainly to the vasculature tissues (Figures 2B–G). Microscopic examination at higher magnification detected the GUS signal in conducting elements that are adjacent to the xylem system, which we speculate to be the phloem or phloem associated cells (Figures 2E,G). In addition, the GUS signal was also observed in the primary root (PR)/lateral root (LR) junctions (Figure 2H) and in the quiescent center (QC, Figure 2H). Interestingly, GUS activity was also detected during osmotic and ABA treatments in hydathodes but not in guard-cells (Figure 3A).

Since osmotic stress responses may be ABA dependent or independent (Yoshida et al., 2014a), we wanted to determine whether the upregulation of *RabA2b* promoter activity during osmotic stress requires ABA. Therefore, we transformed the ABA insensitive mutants: *abi 1-1, abi 2-1, and abi 4-1* with the native *Pro:RabA2b-GUS* construct described above. GUS promoter analysis was then performed as described above in *abi* positive transformants that were ABA treated or osmotically challenged. We observed that strong reduction in the GUS activity occurred in the *abi* transformats background, but not in wt transformats (Figures 3B–D), indicating that ABA is required for regulating *RabA2b* transcription during osmotic stress. Based on these results we mined the *RabA2b* promoter region in the Eukaryotic Promoter Database (EPD)^8^ (Dreos et al., 2015) and identified potential binding motifs for the following ABA responsive transcription factors: ABA insensitive 5 (ABI5), ABA repressor1 (ABR1), Dehydration-responsive element binding protein 3 (DREB3), dehydration-responsive element binding protein 26 (DREB26), Arabidopsis thaliana homeobox protein 16 (ATHB16) and related to Apelata 2.12 (RAP2.12) (Supplementary Figure 2).

### Overexpression of RabA2b in Arabidopsis Increased Drought Resistance

**FIGURE 4:**
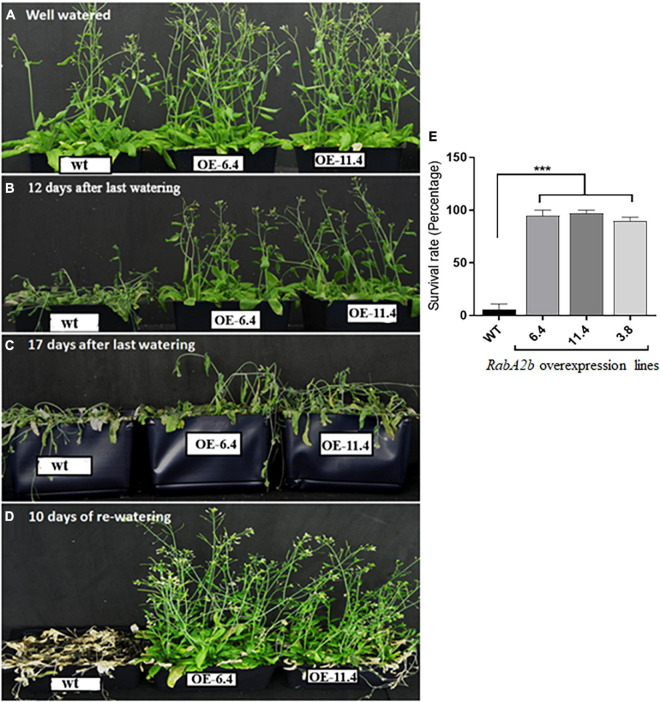
Drought tolerance in RabA2b Overexpressing plants. Transgenic plants constitutively overexpressing *RabA2b* (lines OE 6.4 and OE 11.4) exhibit a significantly high drought tolerant phenotype compare to wild type plants. **(A)** *RabA2b* over-expression and wild type plants were grown under well-watered control conditions for 5 weeks. After 5 weeks, irrigation was withheld for 12 **(B)** and 17 **(C)** days to induce gradual drought to the plants. **(D)** Complete recovery of the overexpressing lines after 10 days of proper irrigation. **(E)** Graphical representation of survival rate of RabA2b over-expressing lines in the drought stress test. Asterisks indicate significant differences between wild-type (wt) and OE plants. All error bars denote the SD, *n* = 24 or 36 plants. *P* < 0.01 (student *t*-test). Experiments were repeated thrice.

To study the potential effect of RabA2b on Arabidopsis performance during water stress, we cloned its transcribed region, fused its N-terminal to GFP (Figure 1C) and overexpressed it in Arabidopsis wt plants (Col-0 background). We then selected T3 homozygous plants (Figure 1D), which appeared normal and resembled the non-transformed wt plants during standard growth conditions (Figure 4A). Nevertheless, during drought conditions, which lasted 18 days, the RabA2b overexpressing lines exhibited a strikingly resistant phenotype: While the wt plants lost their turgor and collapsed 12 Days After Last Irrigation (DALI, Figure 4B), the overexpressing lines remained turgid for an additional 5 days (Figure 4C). Furthermore, when the plants were re-watered on 18 DALI, the wt plants failed to recover and died, whereas the overexpressing lines recovered completely and resumed to bloom and to set seeds (Figure 4D). We further examined homozygous *raba2b-1* T-DNA knockout mutant plants (Figures 1C,D) during the described drought regime and found they behaved similarly to the wt plants (Supplementary Figure 3).

**FIGURE 5:**
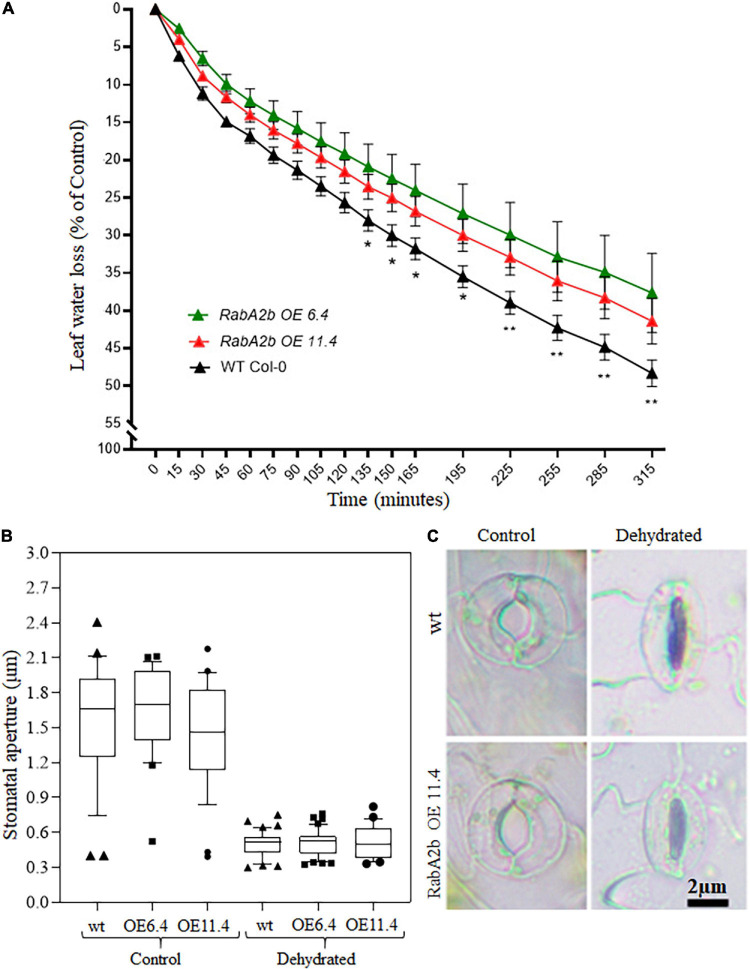
Water loss and stomatal measurements in detached leaves of WT and *RabA2b* over-expressing lines. **(A)** Water loss measurements in detached leaves of wild-type and transgenic plants. Rosette leaves were detached from well irrigated 8 week -old plants and placed on laboratory bench for up to 315 min. Fresh weight Loss was monitored as a function of time, as described in the Section “Materials and Methods.” Shown is a representative triplicate experiment (*n* = 5 leaves from five individual plants ± SE). Two-way ANOVA with Uncorrected Fisher’s LSD multiple comparison post-test, was performed to compare percentage change in initial weight of WT vs. Rab OE 6.4 or WT vs. Rab OE 11.4 at the said time interval. Only significant differences are displayed (*p < 0.05 and **p < 0.006). **(B)** Box plot for stomatal aperture measurements in detached leaves of WT and RabA2b OE lines just after detaching (control) and 120 min after leaf detachment (dehydrated). Dark horizontal lines within the box represent the median, the box representing the 10th and 90th percentiles, the whiskers of 1.5 interquartile range (IQR) limits and outliers are represented by dots/squares/triangles (*n* > 45). **(C)** Representative images of Stomata measured in **(B)**. Micrograph were captured as described in Section “Materials and Methods.”

We further characterized the water loss rates of the tested genotypes by monitored over time (from 0 to 315 min) the reduction in fresh weight of detached leaves, which were removed from well-irrigated plants. We found that the water loss from wt leaves was significantly greater as compared with leaves detached from *RabA2b* OE plants (Figure 5A). To find out whether the moderate water loss observed in the transgenic leaves can be related to a more effective stomatal closure, the stomatal aperture size was measured. Under control conditions of well-irrigated plants (Time 0) the stomatal pore was open similarly in all the genotypes (Figures 5B,C). During 120 min after leaf detachment, the pore size shrank almost completely, however, no significant changes were observed in the stomatal pore size between the tested genotypes (Figures 5B,C). Since the stomatal density per area unit was similar in all the genotypes (Supplementary Figure 4), these results suggests the involvement of other non-stomatal source of water loss, which differed between wt and the transgenic plants.

### Spatial Distribution of RabA2b on Plasma Membrane Ascertained by Confocal Microscopy

**FIGURE 6:**
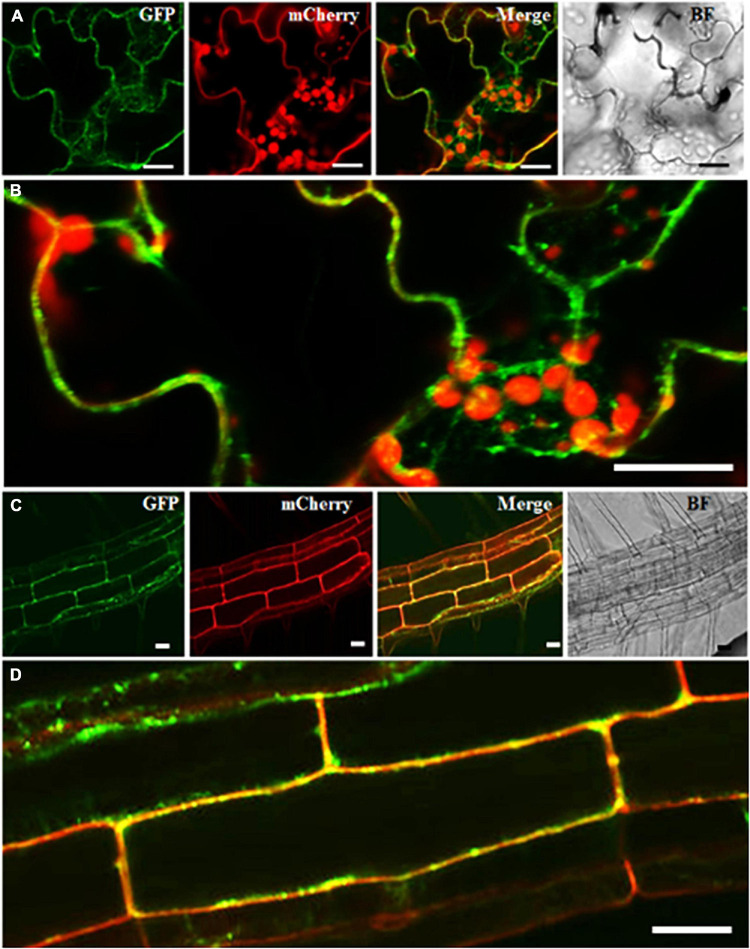
Spatial intra-cellular distribution of RabA2b:GFP. Representative confocal fluorescent microscopy images of double transgenic plants expressing *Pro35S:GFP-RabA2b* and membrane marker aquaporin PIP2a-mCherry. Panels **(A,C)** are the image of leaf epidermis and elongated root cells, respectively, of 5 days old plant showing Rab: GFP signal in green, PIP2A: mCherry signal in red, merge of green and red channel, and bright-field of the focused sample. Panels **(B,D)** are the enlarge area of leaf and root highlighted by white rectangle box showing merge image. Scale bar = 20 μm.

In the past, RabA2 proteins were shown to communicate with the cell-plate and the PM in meristematic root tip cells only (Chow et al., 2008). To validate that RabA2b PM targeting occurred in all cell types of the overexpressing lines, we transformed these lines with a PM specific marker - the *Pro35S:PIP2-mCherry* construct (Nelson et al., 2007) and isolated double transgenic *Pro35S:GFP-RabA2b/Pro35S:PIP2-mCherry* plants. We then performed a subcellular localization study by confocal microscopy. We observed in mature shoot and root cells of these plants substantial overlap between the green (RabA2b) and red (PIP2) fluorescent signals (Figure 6). Further examination of the GFP-RabA2b cytoplasmic signal detected strong co-localization with the Red signal of the endo-membrane marker FM4-64 (Supplementary Figure 5). Nevertheless, not all of the FM 4-64 stained endosomes/vesicles co-localized with the GFP signal (Supplementary Figure 5), indicating the specificity of RabA2b to a certain type of endosomes.

### Plasma Membrane Isolation and Proteomics

**FIGURE 7:**
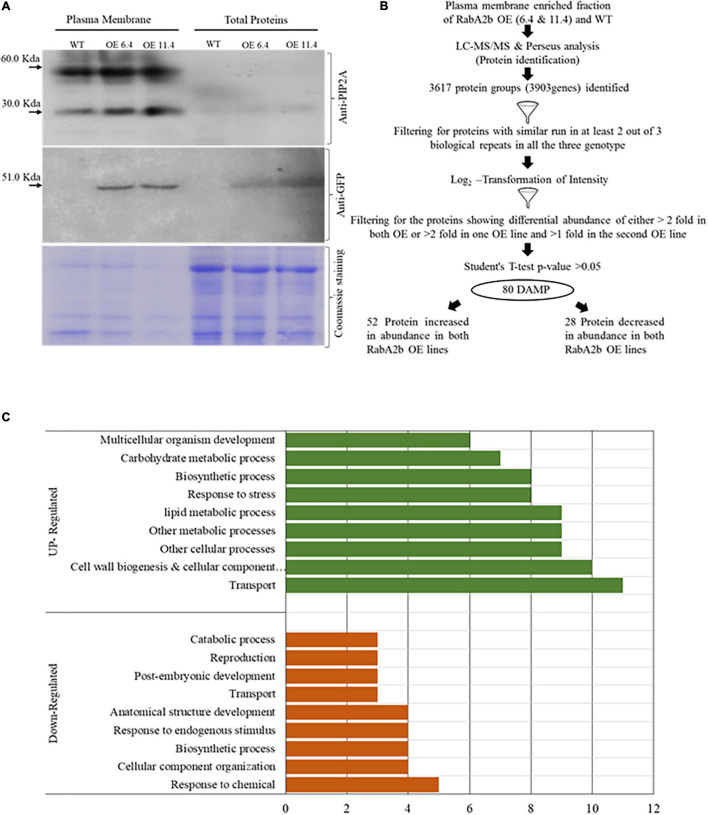
Proteomics of PM enriched fractions in wt and RabA2b overexpressing plants. Total protein and Plasma membrane (PM) enriched fractions were isolated from 11-day old wt and two RabA2b OE lines. The isolated proteins were separated by SDS-PAGE and probed with antibodies against PIP2A and GFP. **(A)** Western blot developed using anti-PIP2A antibody, showing two bands representing the dimeric (52 kDa) and monomeric (30 kDa) forms of PIP2A and 51 kDa band probed by anti-GFP antibody, represents the combined mass of chimeric RabA2b:GFP protein. In addition, shown is Coomassie staining for equal loading of PM-enriched fraction and total protein isolated form mentioned above genotypes. **(B)** Flowchart for the LCMS/MS-based proteomics study of these PM-enriched fractions. The identified protein group were first filtered for their consistent presence or absence in at least 2 out of 3 biological repeats in all three genotypes. The second filter was applied for Log2 transformed intensity. A total of 80 proteins were identified showed differential abundance of either >2 fold in both OE or >2 fold in one OE line and >1 fold in the other OE line. **(C)** Gene Ontology (GO) enrichment analysis of the DAMP for Biological Process in the OE lines. The Bar graph represents the biological functions of the genes corresponding to DAMP proteins with increased (upregulation) and decreased abundance (downregulation). The GO analysis was done using “TAIR GO” annotation search and “AgriGo” https://www.arabidopsis.org/tools/bulk/go/index.jsp.

Based on RabA2b PM targeting (Figure 6 and Supplementary Figure 5), we speculated that overexpressing RabA2b also altered the profile of the PM Proteome that may exhibit stress protective properties. Therefore, we extracted using a two-phase Dextrose-Sucrose gradient, a fraction highly enriched with PM proteins. To study the trafficking effect without the possible interference of drought and osmotic stresses which are known to affect the PM proteome (Wang et al., 2016; Kosová et al., 2018), we sampled plants grown under control conditions. The isolated proteins were exposed to both PIP2 and GFP antibodies to ascertain PM protein enrichment. As compared with the total cell protein samples, these fractions exhibited a high affinity to the PM marker PIP2 antibody (Figure 7A). Notably, the PIP2 Western blot analysis revealed two distinct protein bands corresponding to the monomeric (30 KDa) and dimeric (58 KDa) protein weight as reported previously (Santoni et al., 2003; Abas and Luschnig, 2010) (Figure 7A). Exposure to the anti-GFP antibody confirmed the presence of RabA2b in the samples of the transgenic lines (Figure 7A). A 51.0 KDa band, which matches the predicted size of the chimeric RabA2b:GFP protein we constructed (23 KDa + 28 KDa, the molecular weight of the RabA2b and the GFP proteins, respectively) was observed in the fractions obtained from the overexpressing lines, but not in the wt sample. The GFP signal observed in the PM protein enriched fraction provides another line of evidence to our confocal microscopy study observations which detected significant co-localization between the RabA2b GFP signal and the PIP2 mCherry fluorescence of (Figure 6). Coomassie brilliant blue was used to show equal loading of each sample (Figure 7A).

The PM protein fractions were further analyzed by quantitative label-free proteomics analysis. An equal amount of membrane enriched fractions from each genotype (wt and the RabA2b overexpressing lines OE6.4 and OE11.4) were reduced, alkylated, trypsinized, and subjected to quantitative label-free proteomics analysis (LC-MS/MS). The analysis revealed a total number of 3,617 known protein groups comprising 3,903 genes. The downstream proteomic data analysis is presented by a flow chart (Figure 7B).

Gene Ontology enrichment analysis (agriGO-v2^9^) of these genes for the GO domain cellular component (CC) shows 61 and 35% enrichment of the terms ‘membrane’ and ‘plasma membrane,’ respectively (Supplementary Table 3). Furthermore, 80 protein groups were filtered based on Log2 transform LFQ (label-free quantitation) intensities, showing differential membrane abundance with change of either twofold in both overexpressing (OE) lines or twofold in one OE line and onefold in the second OE line with *p*-value > 0.05 (Figure 7B and Supplementary Table 4). To identify the over represented biological processes (BP) and molecular function (MF) OE samples, the uniProt ID of these differentially- abundant membrane proteins (DAMP) were first converted to gene ID and then analyzed through the “AgriGO and “TAIR GO annotation search”^10^ (Figure 7C and Supplementary Table 5). The genes corresponding to these DAMP were henceforth called differentially regulated gene (DEG). Out of these 80 DAMP, 52 protein showed increased abundance, while other 28 protein showed decreased abundance (Figure 7B).

The genes corresponding these proteins with increased or decreased abundance are called upregulated and downregulated genes respectively (Figure 7C). The enriched BPs in the upregulated group are transport (11 genes), Lipid metabolic process (9 genes), response to stress (8 genes), biosynthetic processes (8 genes), Cell wall biogenesis and cellular component organization (10 genes), other cellular processes (9 genes), other metabolic processes (9 genes), carbohydrate metabolic process (7 genes), and multicellular organism development (6 genes) (Figure 7C). It should be pointed out that some proteins falls into several categories of BPs, which are thus represented more than once. Notably, 22 proteins from this group were predicted to contain a transmembrane helix (TMH) while the other 30 proteins lacked it (Supplementary Table 4).

In the downregulated group, the over-represented BP are catabolic process (3 genes), reproduction (3 genes), transport (3 genes), post embryonic development (3 genes), anatomical structure development (4 genes), biosynthetic process (4 genes), cellular component organization (4 genes), response to chemical (5 genes), and response to endogenous stimulus (4 genes). Interestingly, no stress related BP enrichment was detected in GO analysis of that down regulated group (Figure 7C). We further mined the expression profiles of these 80 DAMP through the “hormone series” of the Bio-Analytic Resource – BAR, and identified 17 ABA responsive genes, five of which overlapped the group of 8 “stress responsive” genes mentioned above (Table 1). We then conducted a literature search and found that most of the “stress related” and/or ABA responsive genes were reported be related with environmental as well as biotic stresses (bacterial and fungal) (Table 1): 17 of these genes were associated with water stress (drought, salt, and osmotic) responses, 2 genes with heat stress and 5 genes showed association with biotic stresses (Table 1).

**TABLE 1:** Differentially expressed stress related genes identified through PM-proteomics of RabA2b over expressing plants.

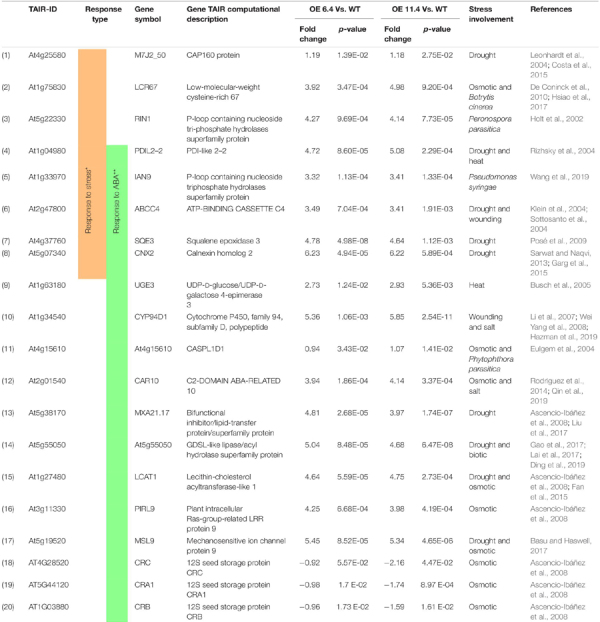

List of 20 differentially abundant stress-related proteins enriched in the membrane of Rab OE plants. Out of 52 proteins (Supplementary Table 4) showing increased abundance in the membrane of *RabA2b* transgenic plant compared to the wt plant, these 17 showed stress relevance either on the basis of their gene ontology information or showed transcript upregulation during ABA treatment (BAR transcriptomic data for stress series). The log2 fold changes between fold OE line 6.4 and 11.4 compared to wt plants were calculated based on extracted ion currents (XICs) of peptides and the *p*-value was determined from a student *t*-test. (*) list of gene with BP “response to stress” identified through GO analysis and (**) Gene Identifies using BAR server.

**FIGURE 8:**
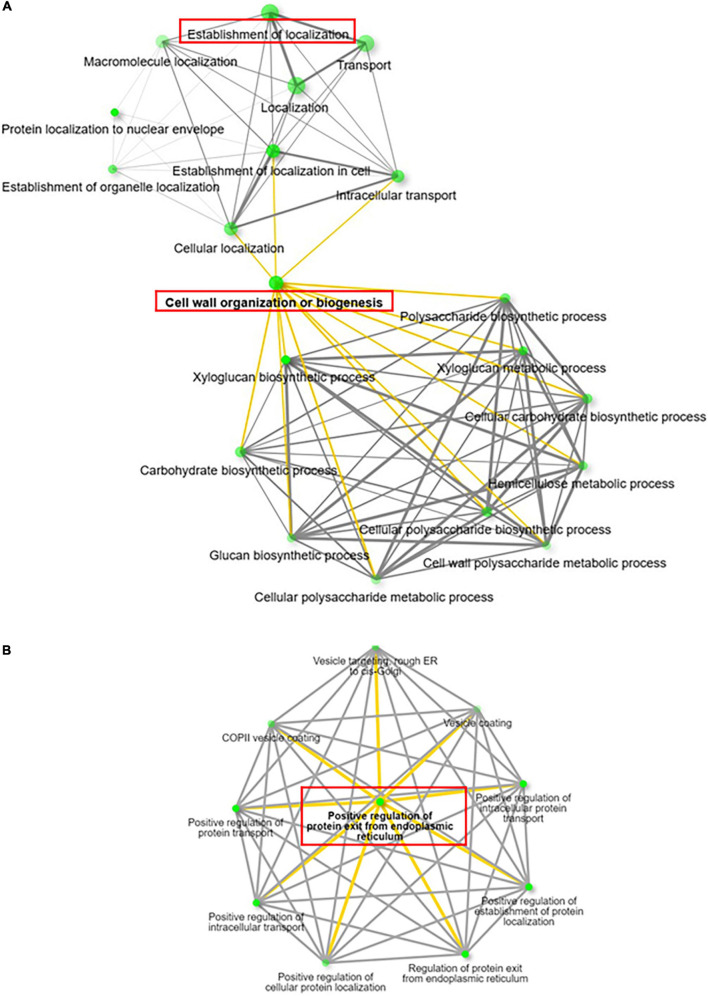
Interconnection of enriched GO terms of up and downregulated genes. Visualization of GO term networks of up **(A)** and down **(B)** regulated genes identified through PM Proteomic analysis using ShinyGo (v0.61) (Ge et al., 2020). Note in **(A)** the enrichment of two major pathways in the RabA2b OE lines: Establishment of localization and Cell wall Biogenesis. In **(B)**, note that the downregulated genes are involved mainly in regulating the trafficking from ER to Golgi. The nodes (Green dots) represent the GO biological processes (BP) while the lines (yellow and gray) represent the interaction between the nodes having a minimum of 20% genes common between two connected BPs.

To further characterize potential interactions among these overrepresented BPs related to these 80 DAMP, we performed enrichment network analysis, of both down/upregulated genes separately, using the ShinyGO tool (Ge et al., 2020). ShinyGO visualizes the interaction between the biological processes through nodes and connecting lines (Figure 8A). The network visualization generated for upregulated genes recognized enhancement of two significant processes in *RabA2b* overexpressing lines, namely “localization” (or “establishment of localization”) and “cell wall biogenesis” (Figure 8A). The BP establishment of localization is defined as any process occurring in a cell that localizes a substance or cellular component, which may occur via movement, tethering or selective degradation (Ashburner et al., 2000). The combined upregulation of these two biological processes suggests therefore that *RabA2b* overexpression leads to an increase in transport of cargo toward the plasma membrane and the cell’s apoplastic region.

Similar ShinyGO analysis which was performed for the downregulated genes showed the overrepresentation of several BPs related to “positive regulation of protein exit from endoplasmic reticulum,” such as Regulation of protein transport, regulation of protein exit from endoplasmic reticulum, COPII vesicle coating, and vesicle targeting- rough ER to *cis*-Golgi. Therefore, indicating that vesicle trafficking from endoplasmic membrane to Golgi was reduced (Figure 8B).

### Cuticle Permeability Assays and RT-qPCRs

**FIGURE 9:**
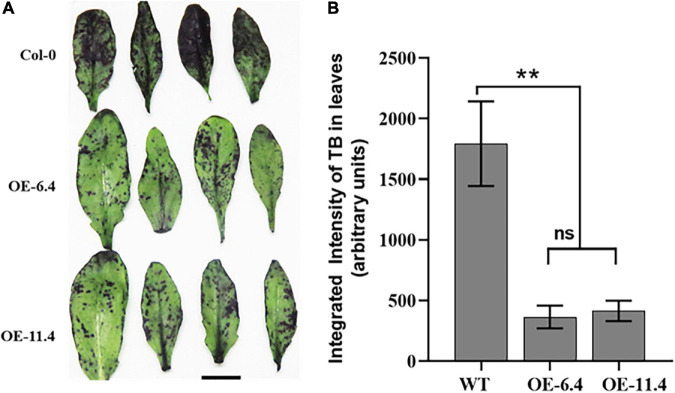
Epidermal permeability assay in leaves of wt and RabA2b overexpressing plants. Rosette and cauline leaves were sampled from 4 weeks old wt or transgenic Arabidopsis plants. The leaves were incubated in aqueous solution of 0.05% (W/V) Toluidine Blue (TB) for 2 h and washed with water. **(A)** Leaves of wt, OE-6.4 and OE-11.4 after TB staining test, **(B)** quantification of the TB staining by imageJ analysis. Two asterisks (**) represents adjusted *P*-values between 0.01 and 0.001. Scale bar = 1 cm.

Since cuticle metabolism related proteins (GDSL, LTP5, and SEQ3) (Arondel et al., 2000; Jetter et al., 2006; Li et al., 2017) were highly abundant in PM fractions of the OE lines (Figure 7C, “Lipid metabolic processes” BP), we suspected that features of the exo-cytoskeleton were altered in these lines. We therefore tested the leaf cuticle permeability in the different genotypes by Toluidine blue (TB) assay, which accumulates inside leaf tissues after crossing the cuticle barrier (Tanaka et al., 2004). As compared with wt, the TB staining in the transgenic leaves was very faint and highly scattered, indicating therefore that their leaf surface is significantly less permeable than the wt (Figure 9). This result is in line with the drought resistant phenotype of the transgenic lines that exhibited enhanced water retention properties compared to the wt (Figures 4, 5A).

Since the differential abundance of the PM proteins in the RabA2b overexpressing lines could potentially arise from altered gene expression, we randomly selected several DE proteins and examined by qRT-PCR their expression profiles in the studied genotypes. We found that the expression of *SQE3* (At4g37760), *GDSL*(At5g55050), *OGHF* (At3g55430), and *ABCC4* (At2g47800) in the overexpressing lines did not differ significantly from their expression in wt (Supplementary Figure 6). Therefore, rather than attributing their high abundance in the PM of the transgenic lines to increase in gene expression, it is more likely that increased PM trafficking occurred in these lines, as suggested by the ShinyGO analysis described above (Figure 8A).

## Discussion

### Transcriptional Regulation of *RabA2b*

Developmental perspectives of RabA2b and its promoter activity have been previously studied in dividing root tip cells (Chow et al., 2008). However, the previous study did not address the potential role that RabA2b might play in response to abiotic stresses. Interestingly, in that study, the 1.605 KB genomic region upstream to *RabA2b*, which was cloned as its putative native promoter, included a fully transcribed gene (At1g07400) with its own promoter. Therefore, the promoter activity results reported in that study might also reflect the activity of other genes beyond RabA2b. To avoid this potential non-specificity, we did not include the full transcribing unit of At1g07400 and excluded its promoter from the region we cloned as *RabA2b* putative promoter (Figure 1C). Examination of the region we cloned revealed that *RabA2b* promoter activity is induced by osmotic stresses as well as by the stress hormone ABA (Figure 2). Moreover, the strong reduction in *RabA2b* promoter activity we observed during osmotic stress in the *abi* mutants background (*abi 1-1*, *abi 2-1*, and *abi 4-1*) indicates that the activation of *RabA2b* during osmotic stress is ABA dependent (Figure 3). Nevertheless, since the RabA2b promoter activity was not totally abolished in the *abi* background, ABA independent pathways may participate to some extent in the *RabA2b* induction during osmotic stress, perhaps *via* crosstalk between ABA dependent and independent pathways which can occur (Yoshida et al., 2014a). Furthermore, *in silico* analysis of *RabA2b* putative native promoter region revealed the presence of multiple predicted biding sites for ABA transcription factors such as ABI5, ABR1, DREB3, DREB26, ATHB16, and RAP212 (Supplementary Figure 2). The latter analysis is in agreement with our results which showed that ABA plays a major role in transcriptional regulation of RabA2b during stress. Future DNA-protein binding assays, will likely identify the exact factors which binds to RabA2b promoter, further shedding light on the ways ABA activates RabA2b transcription during stress.

Since Rab11 proteins are known to be key players in membrane recycling (O’sullivan and Lindsay, 2020), we assumed that during water stress, which leads to membrane damage (Ying et al., 2015; Abid et al., 2018), the activity of *RabA2b* promoter would be observed throughout the plant in most of its cell types. Therefore, it was surprising to find that during osmotic stress, the *ProRabA2b-GUS* signal was restricted mainly to the vasculature, in both the shoot and the root (Figure 2). The GUS signal was detected in conducting elements that are adjacent to the xylem, but not within the xylem (Figures 2E,G). Localization patterns of other genes such as MTK1 (Pommerrenig et al., 2011) and NES-YC3.6 (Shkolnik et al., 2018), which exhibited specific activity in xylem neighboring cells, but not in the xylem, led Pommerrenig et al. (2011) and Shkolnik et al. (2018) to identify these cells as phloem elements. Analogous activity that was restricted to the vasculature-phloem elements, was reported for ABI 4-1 promoter in both the shoot and the root (Shkolnik-Inbar and Bar-Zvi, 2010). Considering the strong reduction of the *ProRabA2b-GUS* signal in the *abi 4-1* background (Figure 3), the similar vasculature expression patterns of ABI 4-1 is in line with our results and support the involvement of ABI 4-1 in RabA2b transcriptional regulation.

Promoter-GUS fusion of several PM residing sucrose transporters such as SUC2 and SUT4 were also shown to exhibit specific vasculature-phloem associated activity (Schulze et al., 2003; Schneidereit et al., 2008). Recently, knockout mutants of SUC2 and other sucrose transporters were reported to exhibit reduced tolerance during ABA mediated water stress (Gong et al., 2015), while additional sucrose transporters were shown to play a protective role in water stress (Xu et al., 2018). Thus, *RabA2b* is possibly involved in trafficking of these transporters or their mediators to the PM during water stress, where their activity can participate in sugar signaling and/or osmotically mediate the water potential and reduce water losses from the water conducting tissues. Similar roles can be postulated for RabA2b involvement with other PM residing proteins known to be involved in water transport, such as PIP water channels that were reported to affect the plant’s tolerance during water stresses (Feng et al., 2018).

Interestingly, we found that osmotic and ABA treatments induced *ProRabA2b-GUS* activity in the hydathode but not in guard cells (Figure 3A). The latter result corresponds with transcriptional information we mined from the BAR microarray database, which shows that RabA2b expression was not induced in the guard cells by exogenous ABA treatments (Supplementary Figure 7). Therefore, during water stress response, RabA2b may be involved in water loss prevention from hydathodes, but is probably not directly involved in mediation of stomatal closure. Thus, although Arabidopsis hydathodes were recently reported t respond to ABA similarly to stomata (Cerutti et al., 2017), the transcriptional regulation of *RabA2*o*b* by ABA seems to differ in each of these cell types.

### Effects of RabA2b Overexpression on Drought Tolerance and Plasma Membrane Proteomics

When RabA2b was overexpressed, a dramatic affect was observed in the transgenic lines, which exhibited significantly improved drought tolerant phenotype (Figure 4). We also tested plants of the *raba2b-1* T-DNA knockout mutant during drought conditions. Based on the inducible nature of RabA2b activity by osmotic stress and ABA, we expected that the knockout will exhibit increased drought sensitivity. Nevertheless, we found that during drought conditions, the *raba2b-1* plants behaved similarly to the wt (Supplementary Figure 3). In the past, due to redundancy, four RabA1 members had to be mutated to demonstrate increased salt sensitive phenotype in the *raba1* quadruple mutant (Asaoka et al., 2013a). It is therefore possible that *RabA2b* is redundant with other RabA2 members such as *RabA2a* and *RabA2c* whose expression was also upregulated during water stress (Supplementary Figure 1A). Note that for this study we chose to examine drought stress. This is in contrast to osmotic stress, chosen for studying promoter activity, as for promoter activity analysis we use young seedlings, which cannot be dried in soil for long durations.

Manipulation of trafficking pathways in Arabidopsis has been shown in the past to affect the profile of PM proteins. For example, disruption of retrograde membrane trafficking to the late Golgi in the *hit1* mutant (Lee et al., 2006) resulted in altered profile of PM proteins (Wang et al., 2011a) and reduced the plant’s ability to withstand heat stress. However, these previous studies did not resolve the exact identity of the proteins that were altered in the PM of that mutant. Moreover, to our knowledge, it has not been previously shown that overexpression of RabA members alters the PM composition (lipidome or proteome). Our findings shed light on possible pathways involved in the observed increased drought tolerance. We validated by confocal microscopy that RabA2b targeted the PM in different cell types of the transgenic lines (Figure 6). Next, we isolated PM enriched fractions from wt and RabA2b overexpressing lines, which were further confirmed by a specific PM marker – the PIP2 antibody (Figure 7A). The colocalization of RabA2b with PM proteins indicated that it may be involved in the trafficking of several proteins to the PM. To examine this possibility we adopted a proteomic approach and identified in these fractions 80 proteins, which were differentially abundant in the PM samples of RabA2b overexpressing lines compared to the wt PM samples (Figure 7B). We identified 8 proteins that were stress related and another 17 proteins whose transcript was highly responsive to the stress hormone ABA (Figure 7C and Table 1), which are supported by the literature to be involved with water stresses, such as drought, osmotic stress and salinity as well as connection to heat stress and pathogenicity. For example, SQE3 (squalene epoxidase 3, At4g37760) plays a major role in sterol biosynthesis in Arabidopsis shoots. SQE3 was suggested to possess overlapping functions with SQE1, whose knockout mutants were shown to be highly sensitive to drought (Posé et al., 2009). In addition, *CASPL1D1* (Casparian strip membrane domain protein-like D1, At4g15610) which was exclusively expressed in suberized endodermal cells and interacted with PIP2;1 was proposed to be involved in water transport regulation (Champeyroux et al., 2019).

Furthermore, knockout mutants of *ABCC4* (ATP binding cassette, At2g47800) which encodes an ABC transporter, were shown to exhibit increased sensitivity during drought conditions due to impaired stomatal closure (Klein et al., 2004). Another example, *CAR10* (C2-domain ABA-related At2g01540), is a member of the CAR family which is reported to mediate the recruitment of PYR/PYL ABA receptors to the plasma membrane (Rodriguez et al., 2014). Overexpressing CAR genes in Arabidopsis increased the plant’s sensitivity to ABA (Yoshida et al., 2014b). Recently, it was reported that enhancing the stability of CAR9 or CAR10 proteins in Arabidopsis improved the plant’s drought tolerance (Qin et al., 2019). Therefore, it is possible that the increased abundance of CAR10 in the PM of RabA2b overexpressing lines, enhances indirectly the perception of ABA, through increased availability of ABA receptors.

Drought resistance has been altered in other plant species by manipulating several similar gene family members or homologous genes to our identified water stress responding genes: GDSL-like Lipases are known to play a role in Cutin metabolism and extracellular exportation (Girard et al., 2012). One of the water stress responding proteins is a member of *GDSL-like Lipases/Acyl hydrolase* superfamily (At5g55050). A Recent report showed in Barley that GDSL-esterase/acyltransferase/lipase mutant (CER-ZV) exhibited reduced leaf cuticle and increased drought sensitivity (Lai et al., 2017). Moreover, *CNX* (Calnexin homolog 2 At5g07340) is a highly conserved endoplasmic reticulum (ER) chaperone protein (Liu et al., 2017). Overexpressing the *OsCNX* rice homolog in tobacco enhanced the plant’s tolerance to dehydration stress conditions (Sarwat and Naqvi, 2013).

Taken together, the abundance of these water stress responsive and/or drought tolerance inducing proteins in the PM fractions of RabA2b overexpressing lines (and their absence from wt) is in line with the improved drought resistance of the corresponding phenotypes. Therefore, it can be proposed that the RabA2b overexpressing plants are more primed to drought than the wt plants.

Interestingly, we also identified 5 proteins that respond to various biotic stresses (Table 1). Considering the earlier reports about the role of RabA proteins in plant-pathogen interactions (Choi et al., 2013; Ellinger et al., 2014; Leborgne-Castel and Bouhidel, 2014), it is possible that the RabA2b overexpressing lines will show in future studies phenotypes that possess altered resistance to fungal or bacterial infections.

The ShinyGO enrichment network analysis of the identified 52 increased abundance proteins, recognized enhancement of two biological processes: establishment of localization and Cell wall biogenesis (Figure 8A). The juxtaposition of these two biological processes suggests that RabA2b is involved in increasing the transport of cargo toward the plasma membrane and the cell’s apoplastic region, in the *RabA2b* overexpressing lines. This suggestion is in line with the current knowledge about the active involvement of RabA proteins in PM and cell wall trafficking (Ebine and Ueda, 2015; Nielsen, 2020). Indeed, 9 cell-wall-processing proteins and 5 extracellular lipid metabolism proteins (GO: 0005576 – At5g55050, At1g75830, At4g377760, At1g27480, and At3g51600), were overrepresented in the PM fractions we isolated from the RabA2b OE lines (Supplementary Table 4). In the latter group, three proteins (GDSL, LTP5, and SEQ3) are related to cuticle metabolism (Arondel et al., 2000; Jetter et al., 2006; Li et al., 2017). Our cuticle permeability tests by Toluidine blue staining, showed that the transgenic leaves were significantly less permeable than wt leaves (Figure 9). Thus, minimized cuticular water loss is suggested for these plants, which is in line with their improved drought resistance phenotype (Figure 4). Indeed, the increase in cuticle processing proteins mentioned above correlate with increased drought tolerance, as previously described (Yeats and Rose, 2013). This data also correlates with a recent report mentioned above where reduced leaf cuticle was observed in a barley *GDSL-esterase/acyltransferase/lipase* mutant (*cer-zv*), which was found to be drought sensitive (Li et al., 2017). Interestingly, among the DAMPs of RabA2b overexpressing lines, we identified Guanine nucleotide exchange factor *VPS9a* (AT3G19770) which can activate all Rab5 members to GTP-bound forms *in vitro* (Goh et al., 2007). It is therefore possible that in the background of our transgenic lines, its increased abundance facilitates nucleotide exchange with Rab members other than *RabA2b*, resulting in additional enhancement Rab mediated trafficking pathways during drought response.

Among the 28 genes showing downregulation (decreased protein abundance), 3 transport-related genes *SEC16A* homolog (AT5G47480), GTP-binding protein *SAR1A* (AT4G02080), and *SAR1B* (AT1G09180) are known to be involved in protein transport from the endoplasmic reticulum to the Golgi apparatus (Figure 7C and Table 1). Indeed, the ShinyGo analysis performed for this group indicated a decline in the trafficking from ER to Golgi (Figure 8B). This decline may arise due to compensation for RabA2b overexpression which is preferentially intensifying the trafficking toward the plasma membrane and building up competition for existing GTP pool and other trafficking machinery. Interestingly more than twofold decrease was observed in the abundance of RabA2c (Supplementary Table 5), which indicates compensation between RabA2 family members.

Manipulations of trafficking genes, which mediate various intracellular pathways in mammals and plants, can also effect gene expression (Tripathy et al., 2017). However, the transcript levels of several increased abundance proteins which were determined by qRT-PCR, were statistically similar in all the tested genotypes. Therefore, the abundance of these proteins in the PM fractions of the OE lines can be related to the increase in PM RabA2b mediated trafficking. Among the 52 increased abundance proteins, 22 of which were predicted to contain TMH (Transmembrane helix), are likely trafficked to the PM on the vesicle membrane. However, the other 30 increased abundance proteins that lacks the TMH, are probably trafficked to the PM, either as cargo inside these vesicles or through associations with other vesicle membrane bound proteins.

**FIGURE 10:**
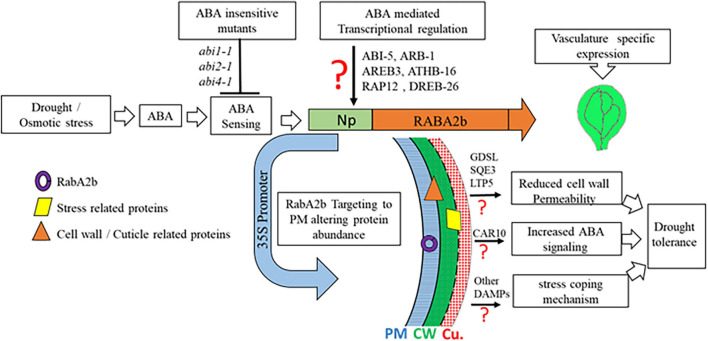
RabA2b modes of action during water stress events. Under drought/osmotic stress ABA-insensitive mutants (*abi1-1*, *abi2-1*, and *abi4-1*) shows a minimal expression of *RabA2b* gene which supports the fact that TF-binding to *RabA2b* promoter is facilitated by the family of ABA responsive adaptor proteins such as ABI1, ABI3, and ABI4. ABA induced expression of *RabA2b* is predicted to be mediated by an unidentified ABA-responsive transcription factors (TFs) binding to predicted TF-motifs present in its promoter. However, the plants over-expressing *RabA2b* under constitutive promoter (CaMV-35S promoter) shows enhanced drought-tolerant phenotype. The microscopic and proteomic studies suggest RabA2b enhance vesicle trafficking toward plasma-membrane (PM) which altered protein localization in the PM/apoplastic region of GDSL, SQE3, and LTP5 resulting in a customized cell wall and cuticle. Nevertheless, their direct role in cuticle modifications needs further validation. These changes in the OE lines modulate surface permeability and prevent non-stomatal water loss, making these plants more tolerant to drought stress. In addition, increased RabA2b trafficking increasing the abundance of CAR10, which is known to facilitate the recruitment of ABA receptor on plasma-membrane. Therefore, it can be speculated that the RabA2b OE plant possess improved ABA sensing or signaling. Apart from these mentioned protein, several other protein identified in the proteomics studies have been reported to provide drought tolerant phenotype (Table 1) therefore possibly playing a role in drought resistance through other unknown stress coping pathways. CU, cuticle; PM, plasma-membrane; CW, cell wall, red question mark (?) represents the need of further confirmations.

In summary, we provide here novel insights into the transcriptional regulation of RabA2b by ABA during the response to water stresses. In addition we show that overexpression of RabA2b in Arabidopsis altered the PM proteome of the transgenic lines, which was enriched with water stress related proteins potentially involved in regulating drought tolerance. We also identified several lipid metabolism and cuticle metabolism related proteins that are capable of affecting cuticle permeability, leading to a less permeable cuticle in the transgenic plants. Overall we demonstrated that the overexpression of RabA2b improved profoundly the plant’s resistance to drought. Our inferred RabA2b mode of action during water stress that leads to enhanced drought resistance is schematically summarized in Figure 10. Further studies of RabA2b trafficking pathways in crops, hold the potential to provide new methodologies for improving drought resistance, which are necessary for coping with the increased climate change challenges (Mora et al., 2018).

## Data Availability Statement

The mass spectrometry proteomics datasets presented in this study can be found in online repositories via ProteomeXchange Consortium in the PRIDE partner repository - http://www.ebi.ac.uk/pride with identifier PXD028140.

## Author Contributions

YL and VA planned and designed the research. VA and IM performed the experiments and analyzed the data. DT counted stomata. YL and VA wrote the manuscript. All authors contributed to the article and approved the submitted version.

## Conflict of Interest

The authors declare that the research was conducted in the absence of any commercial or financial relationships that could be construed as a potential conflict of interest.

## Publisher’s Note

All claims expressed in this article are solely those of the authors and do not necessarily represent those of their affiliated organizations, or those of the publisher, the editors and the reviewers. Any product that may be evaluated in this article, or claim that may be made by its manufacturer, is not guaranteed or endorsed by the publisher.

## Footnotes

1
https://abrc.osu.edu/

2
http://arabidopsis.info/

3
https://www.gabi-kat.de

4
https://www.ncbi.nlm.nih.gov/

5
https://imagej.nih.gov/ij/

6
https://www.maxquant.org/

7
http://www.bar.utoronto.ca/

8
http://epd.vital-it.ch

9
http://systemsbiology.cau.edu.cn/agriGOv2/

10
https://www.arabidopsis.org/tools/bulk/go/index.jsp
