# Supplementary material for: RabA2b Overexpression Alters the Plasma-Membrane Proteome and Improves Drought Tolerance in Arabidopsis
Source: Front Plant Sci. 2021 Oct 6;12:738694. doi: 10.3389/fpls.2021.738694 (PMC8526897; doi:10.3389/fpls.2021.738694)
Supplement: Supplementary file 3 [file Data_Sheet_1.PDF]

Supplementary material

RabA2b mediated plasma membrane trafficking improves water stress tolerance in Arabidopsis.

Vivek Ambastha, Ifat Matityahu, Dafna Tidhar and Yehoram Leshem*

Table of Content	Page no.
Methods	2-3
References	3
Figure S1	4
Figure S2	5
Figure S3	6
Figure S4	7
Figure S5	8
Figure S6	9
Figure S7	10
Table S1	11
Table S2	12
Table S2	13

Methods for Supplementary data

Insilco gene expression

The electronic Fluorescent Pictograph (eFP) and electronic northern browser was used extract RabA2b expression pattern during developmental and under abiotic stresses respectively using the default setting of these programs (http://www.bar.utoronto.ca/).(Toufighi et al., 2005; Winter et al., 2007)

Promoter Analysis for binding motifs of ABA-responsive transcription factors

The "atEPDnew" tool of Eukaryotic Promoter Database (http://epd.vital-it.ch) (EPD) (Dreos et al., 2015) with a search tool for Transcription factor (TF) motifs contained in JASPAR core 2018 plant library was used to for promoter analysis. 1kb sequence (*p:RabA2b*) upstream of translation start site (TSS) of the RabA2b was scanned at a cutoff p-value of 0.0001 and 0.00001 to search binding motifs of ABA-responsive transcription factors (TFs) (Nakashima and Yamaguchi-Shinozaki, 2013; Banerjee and Roychoudhury, 2017)

RNA Isolation, cDNA Synthesis, and Real-Time qPCR.

Total RNA was isolated from MS grown, seven days old seedlings (~100 mg tissue) using spectrum plant total RNA kit (Sigma-Aldrich STRN-50) and 1µg RNA was used to prepare cDNA using Verso cDNA synthesis kit (Thermo-scientific –AB-1453/B). The RT-qPCR was carried on Rotor Gene-6000 (Corbett-Mortlake, NSW, Australia). Data presented is the mean value for three biological and three technical replicates performed for every gene of interest (GOI) and absolute quantification was performed by constructing a standard curve (Udvardi et al., 2008) where the PP2A gene (AT1G69960) was used as an internal control. The details of the primers set used for RT-qPCR in this study are listed in Supplementary Table 1.

FM4-64 Staining

A vail of FM4-64 (Invitrogen, Cat no T13320) containing 100µg dye was dissolved in DMSO to make a stock concentration of 2mM. Root were incubated in RT for 15 min in water containing 2µM of FM4-64 .excitation laser was set at 555 nm, and the preset emission wavelength was used.

Stomata Count

To compare the stomata count in wt and OE lines, the impression replica of the abaxial leaf was made from the Dental resin, Xantopren L-Blue (polysiloxane), and universal plus activator (4:1). The Imprints of abaxial surfaces of rosette leaves from five-week-old Arabidopsis wild-type and RabA2b overexpressing lines were prepared using as described by (Groll et al., 2002). The final surface imprint was prepared by using nail polish on resin replica of leaf and place on glass slides. Imprints from wt and *RabA2b* OE lines were analyzed with Motic AE2000 inverted microscope under 10X lenses fitted with Olympus SC180 camera. Counting was performed on the leaves from 15 plants of each genotype. For each leaf, twelve separate fields of area 454µm x 454 µm were analyzed. For each leaf, twelve separate fields of area 454 µm x 454 µm were analyzed.

References

Banerjee A, Roychoudhury A (2017) Abscisic-acid-dependent basic leucine zipper (bZIP) transcription factors in plant abiotic stress. Protoplasma **254**: 3–16

Dreos R, Ambrosini G, Périer RC, Bucher P (2015) The Eukaryotic Promoter Database: expansion of EPDnew and new promoter analysis tools. Nucleic Acids Res **43**: D92–D96

Groll U von, Berger D, Altmann T (2002) The Subtilisin-Like Serine Protease SDD1 Mediates Cell-to-Cell Signaling during Arabidopsis Stomatal Development. Plant Cell **14**: 1527

Nakashima K, Yamaguchi-Shinozaki K (2013) ABA signaling in stress-response and seed development. Plant Cell Rep **32**: 959–970

Toufighi K, Brady SM, Austin R, Ly E, Provart NJ (2005) The Botany Array Resource: e-Northerns, Expression Angling, and promoter analyses. Plant J **43**: 153–163

Udvardi MK, Czechowski T, Scheible WR (2008) Eleven golden rules of quantitative RT-PCR. Plant Cell **20**: 1736–1737

Winter D, Vinegar B, Nahal H, Ammar R, Wilson G V., Provart NJ (2007) An “Electronic Fluorescent Pictograph” Browser for Exploring and Analyzing Large-Scale Biological Data Sets. PLoS One **2**: e718

(A)

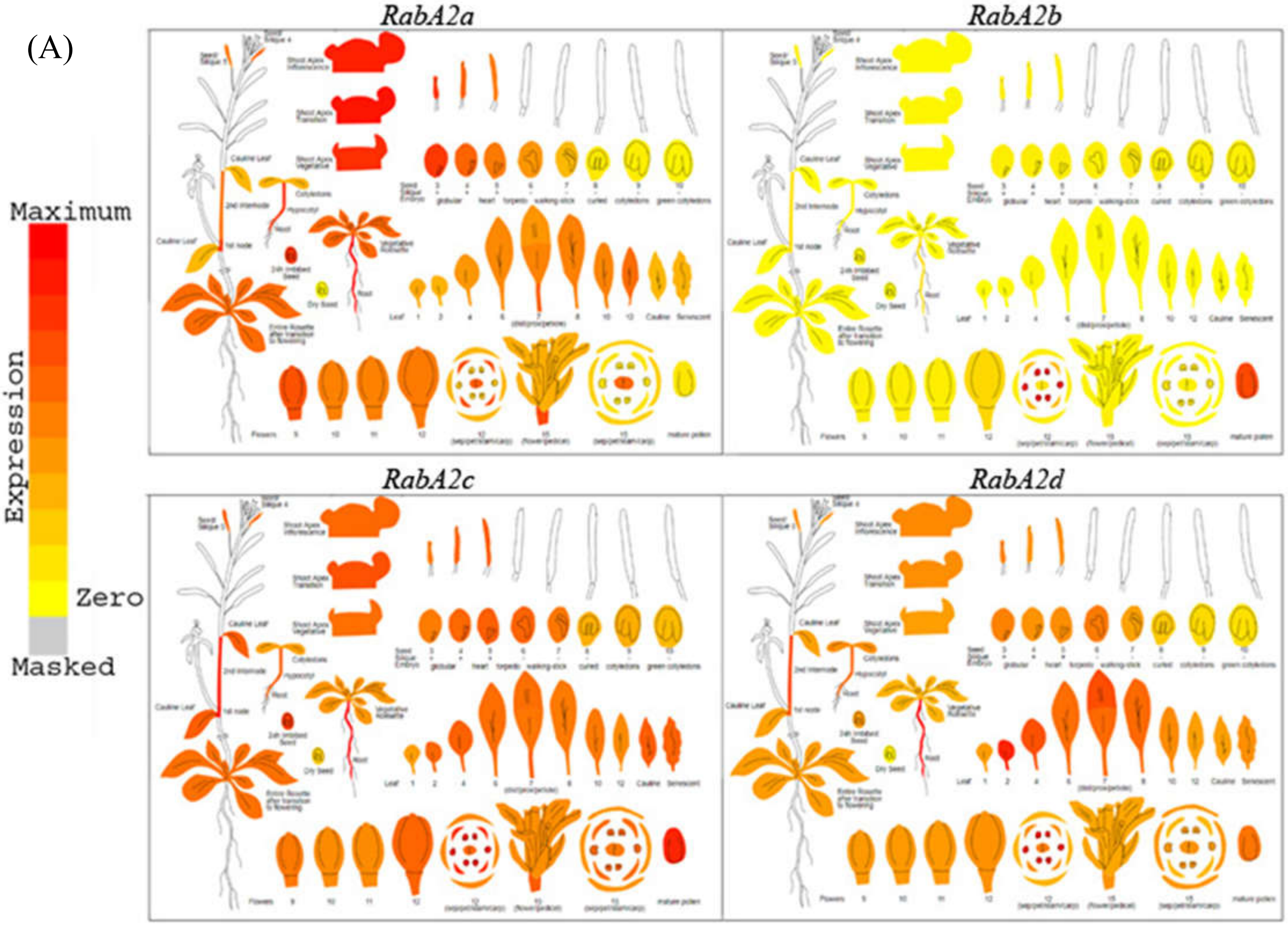

(B)

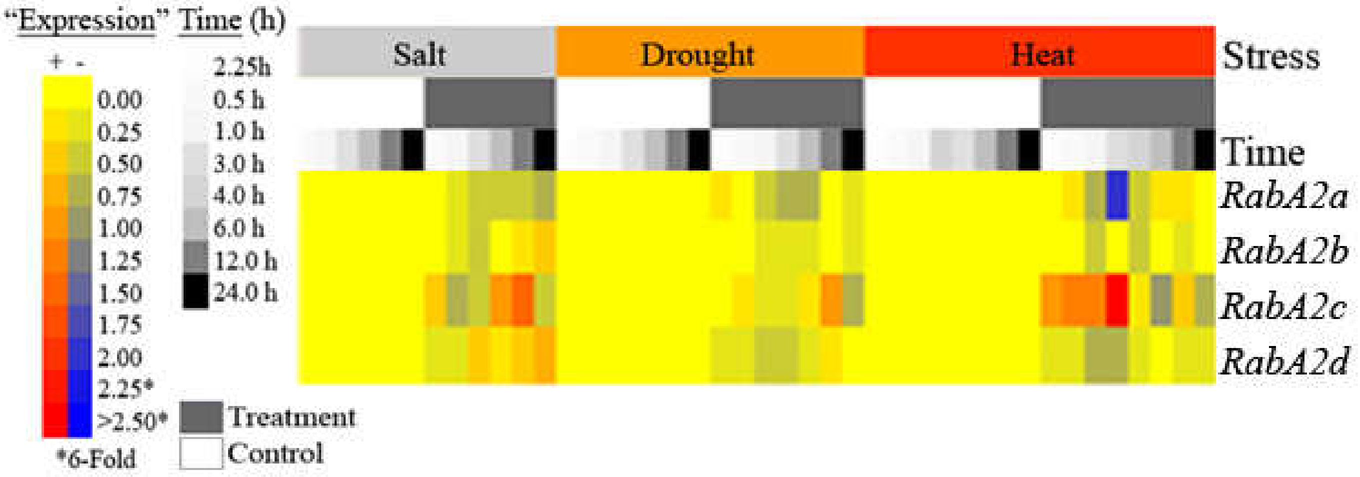

Figure S1- Developmental and environmental expression patterns of the RabA2 sub-family in Arabidopsis. (A) Gene expression of RabA2 family (*RabA2a, RabA2b, RabA2c* and *RabA2d*) during different developmental stages. (B) Gene expression of RabA2 family during several abiotic stresses. The data in (A) and (B) are extracted from the Bio-Analytic Resource – BAR eFP and eNorthen browsers, respectively (https://www.bar.utoronto.ca/). Color coding in (B) indicates the treatment details and the levels of gene expression.

A

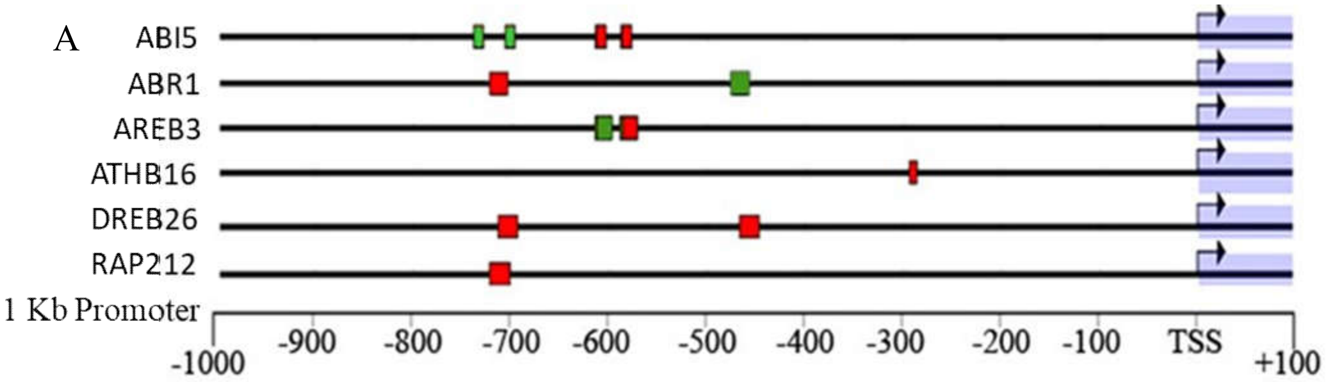

B

TF	p-Value	TF binding motif location in Promoter					
		1st site	2nd site	3rd site	4th site	5th site	6th site
ABI5	0.0001	-738	-706	-614	-613	-588	-587
	0.00001	-614	-588	-587			
ABR1	0.0001	-722	-476				
	0.00001	-722					
AREB3	0.0001	-615	-612	-589	-586		
	0.00001	-589					
ATHB-16	0.0001	-294					
	0.00001	-294					
DREB26	0.0001	-713	-467				
	0.00001	-713	-467				
RAP212	0.0001	-722					
	0.00001	-722					

Figure S2 - Putative binding motifs of ABA responsive transcription factors (TF) in *RabA2b* Promoter : The Insilco analysis using Motif libraries from the JASPAR database predicts (at p value 0.0001 and 0.00001) presence of several cis-acting Elements distributed within +100 and -1000 bp from TSS. (A) The green and red boxes shows the putative location of the respective TF at p-Value 0.0001 and 0.10000 respectively and the arrow in blue rectangle marks the Transcription start site(TSS). The Table (B) shows the exact nucleotide position of these TF binding motifs on the promoter region at p-Value 0.0001 and 0.10000 (https://epd.epfl.ch/arabidopsis/arabidopsis_database.php?db=arabidopsis).

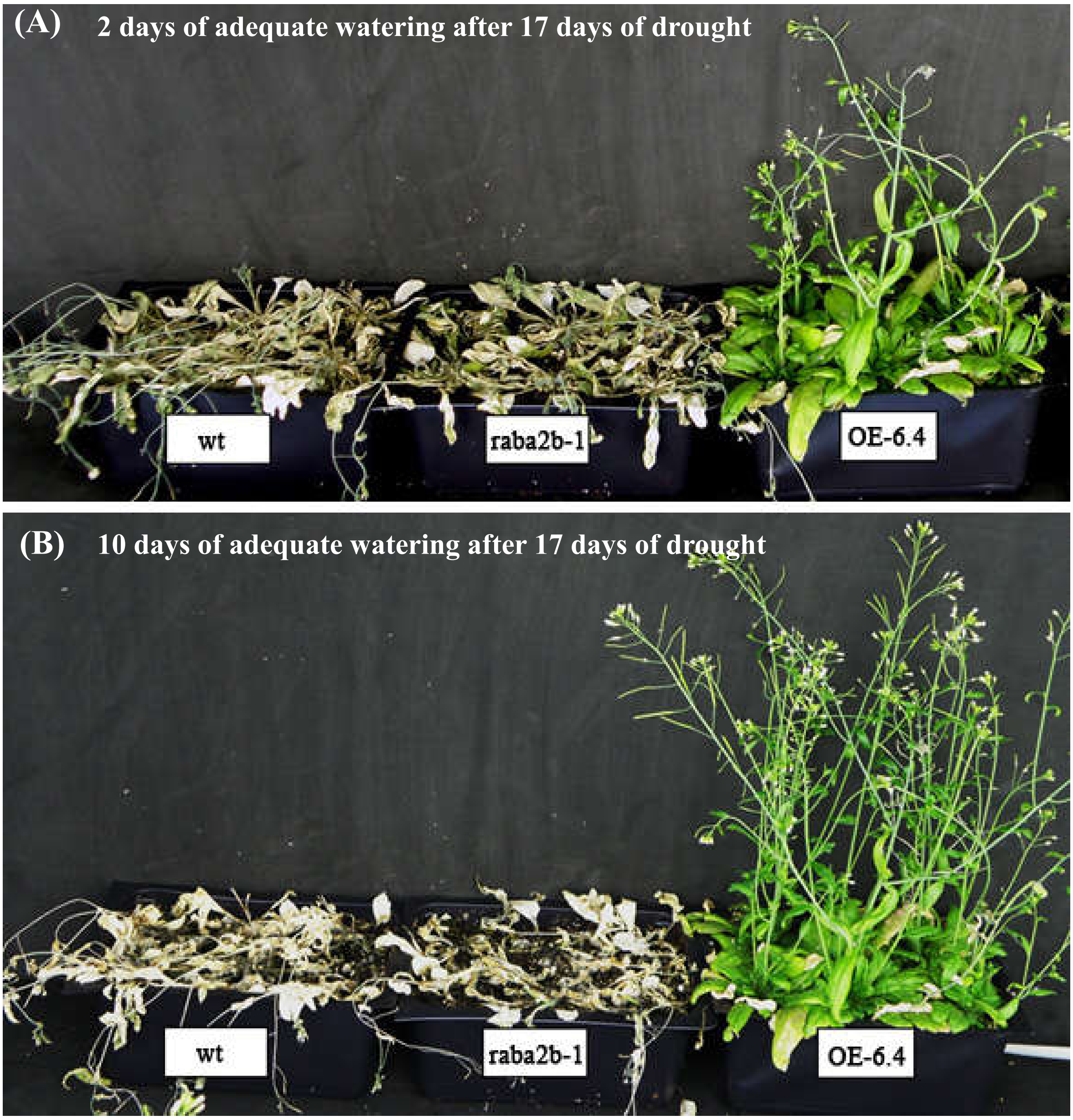

Figure S3 – Drought phenotype of WT Col-0 and RabA2b T-DNA knock line after prolonged water stress. Representative images of 5 weeks old WT Col-0, *rabA2b-1* and *RabA2b* OE-6.4 lines subjected to severs water stress followed by adequate watering for 2 days (A) and 10 days (B) respectively. WT Col-0 and *RabA2b* T-DNA knock down lines fails to recover after prolonged water stress while RabA2b OE lines shows complete drought recovery. All these shown pots were placed together in the same tray and received the similar treatment throughout the experiments. n= 18, the experiment was repeated thrice.

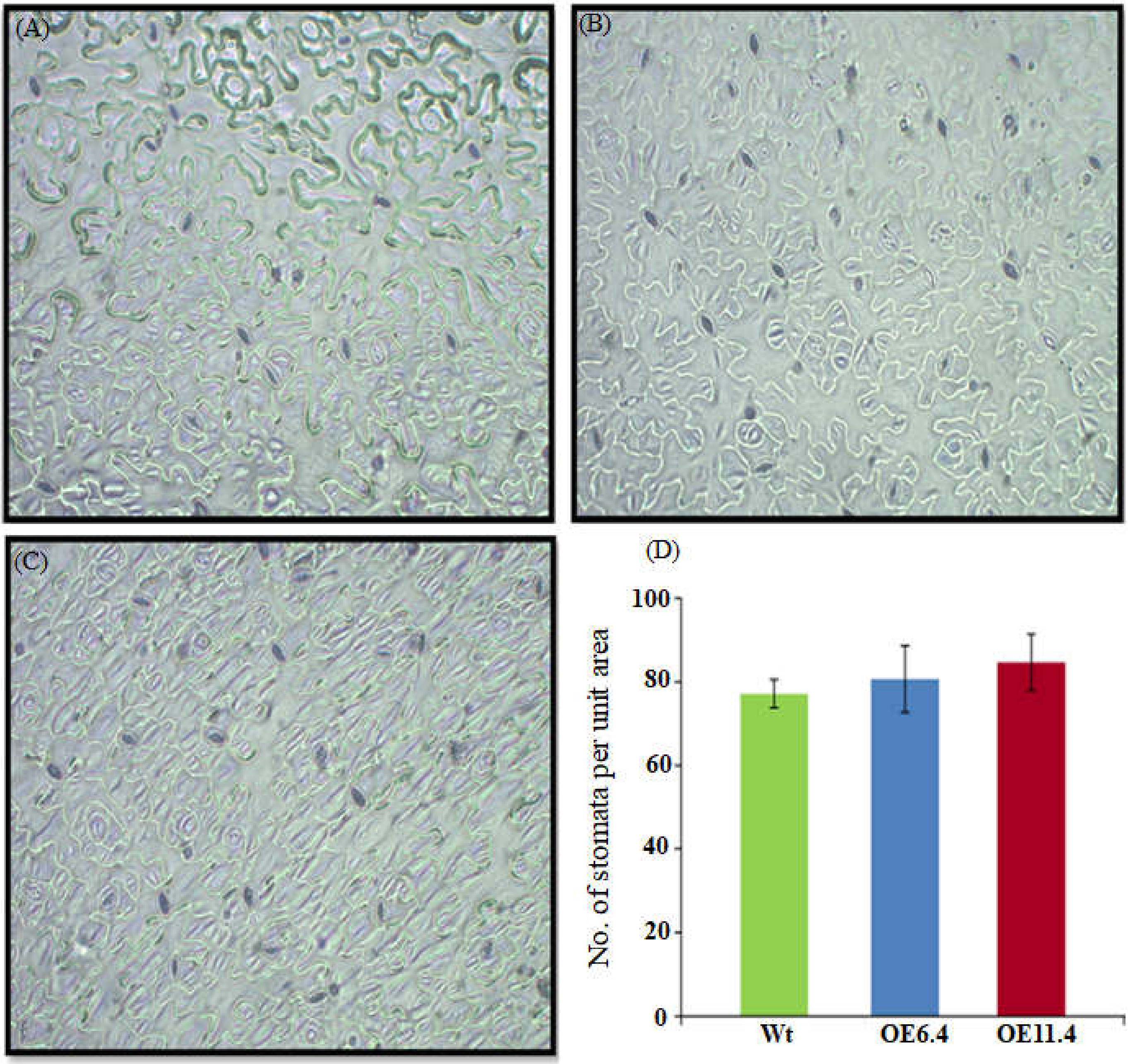

Figure S4. Stomatal density in wt and *RabA2b* OE lines. The stromal density per unit area of wt and Rab OE lines was calculated by preparing silicon leaf replica and imprinting it on glass slides using a white nail polish. Shown are the micrograph of leaf area (454 µm x 454 µm) from (A) wt (B) *RabA2B* OE6.4 and (C) *RabA2b* OE 11.4 as observed under 10X objective. (D) The graph represent the stomatal count of wt. RabA2b and OE 6.4 lines. Counting was preformed on the leaves from 15 plants. For each leaf, twelve separate fields of area 454µm x 454 µm were analyzed.

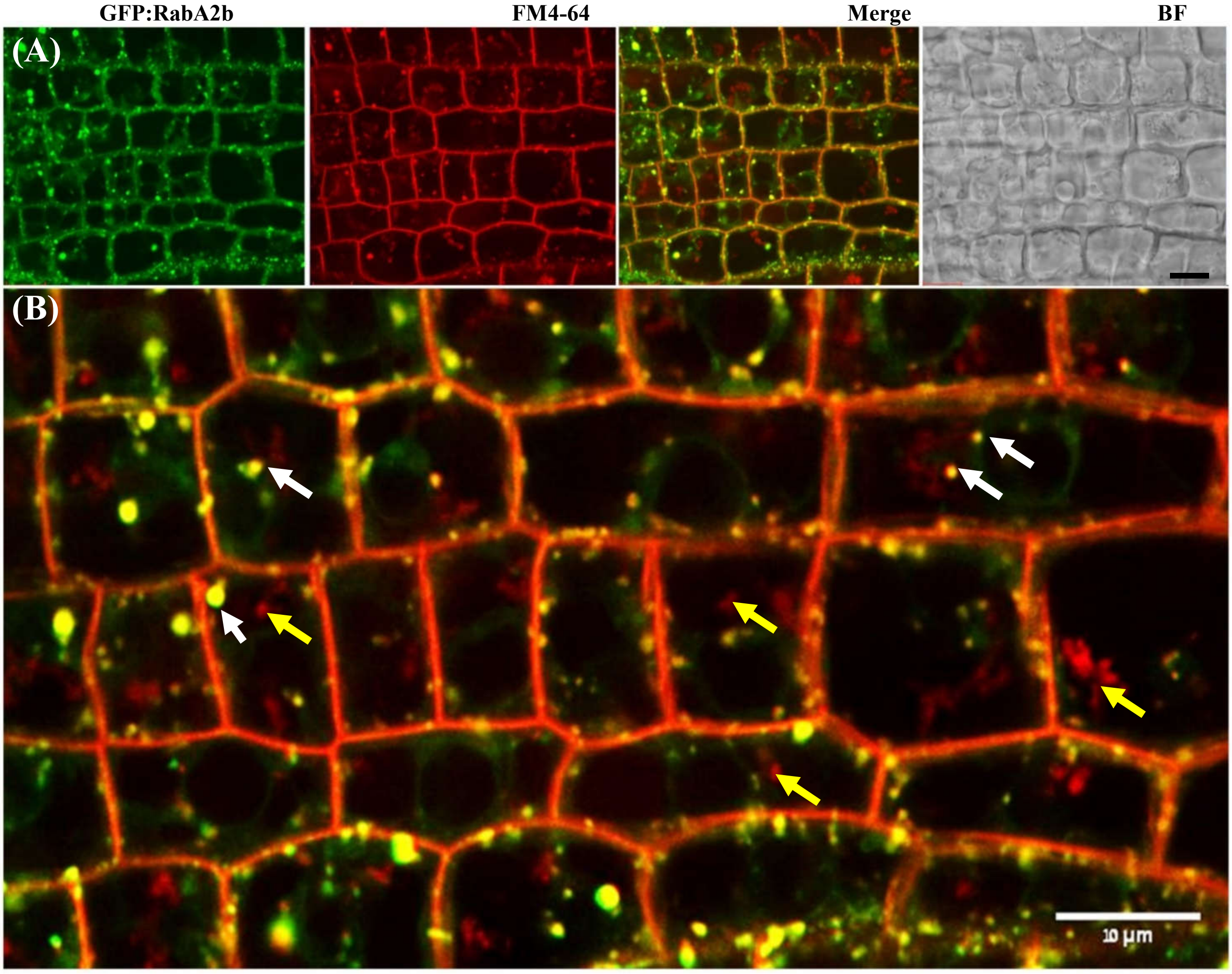

Figure S5 – Sub-cellular localization of RabA2b in root cells.
Confocal Microscopic study was conducted in roots cells of 35S::GFP:RabA2b lines stained with the endosomal marker FM4-64 as described in methods. The Upper Panel (A) shows representative individual channel micrographs of roots cells of five days old transgenic plants. The lower panel (B) is enlargement of the merged image in A.Note that most but not all of RabA2b marked vesicles are co-localized with endosomes and are distributed equally in the cytoplasm and plasm membrane. Yellow arrows marks only FM 4-64 labelled endosomes while the white arrows indicates co-localization of GFP:RabA2b with FM 4-64 labeled endosomes. Scale bar =10µm.

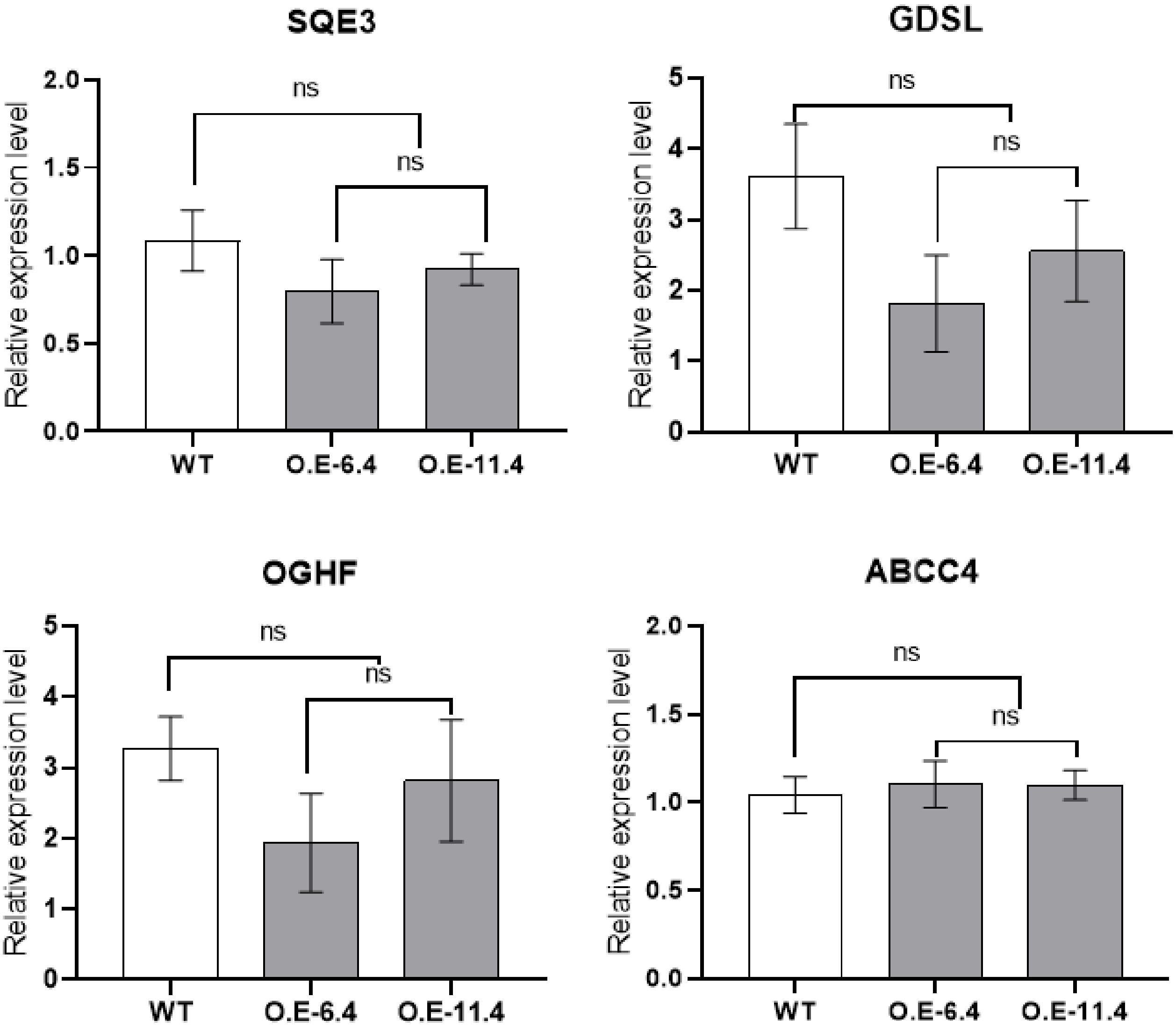

Figure S6 – Gene expression analysis of several proteins highly abundant in PM of RabA2b overexpressing plants.

Real-time RT-PCR analysis of upregulated lipid metabolism and stress related genes, *GDSL SQE3, OGHF* and *ABRCC4* identified through membrane proteomics *RabA2b* over-expression lines. Relative expression levels were calculated in comparison with PP2A. Shown is average values ± SE obtained for three independent biological repeats and (ns) indicate non-significant differences.

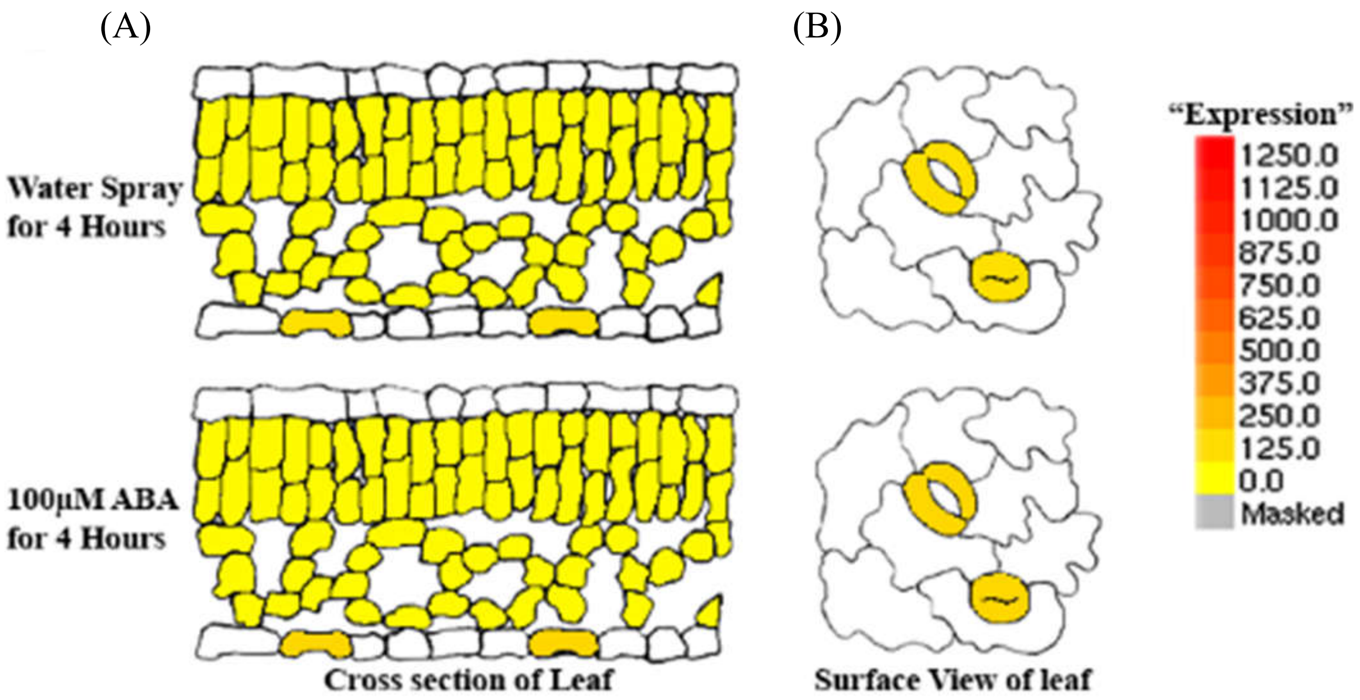

Figure S7 - *RabA2b* expression is not induced in the guard cells by ABA treatments.
RabA2b expression in Guard and mesophyll cell remain unchanged in response to exogenously applied ABA. Shown is the RabA2b expression profile extracted through **Arabidopsis eFP Browser** (http://bar.utoronto.ca/). Transverse cross section (A) and surface (B) view of the leaf, during control and ABA treatment. The color key on the right side indicates the levels of RabA2b expression.

Table S1: The list of primer used in used in this study.

	Primers Name	Primer sequence (5' → 3')	Gene ID
**Primer for genotyping of RabA2b GABI T-DNA mutant lines**			
1	LB1-GABI-Kart	ATAATAACGCTGCGGACATCTACATTTT	
2	Gabi LP RabA2b	GAAAAACAAAGACCCGGAAAG	At1G07410
3	Gabi Rp RabA2b	GTTTCGATTTGCTGCTTTTTG	At1G07410
**Primers used for cloning the RabA2b gene and its promoter**			
1	FP_ Promoter RabA2b	CACCAACATCGGCGATAACGAA	At1G07410
2	RP_ PromoterRabA2b	TGCTTCTCAAATCCTAGCGTC	At1G07410
3	FP_Gene_RabA2b	CACCATGGCGAATAGAATAGATCATGA GTACG	At1G07410
4	RP_Gene_RabA2b	TCTTCCACCTCCCTAGTG	At1G07410
**Primers for Real Time qPCR**			
1	At QPP2A Fw	TGTTGGAGCCCCAGGACTGT	At1G69960
2	At QPP2A Rev	CGTCCTAGTTGGCTCCGGTC	At1G69960
3	RabA2B Fw	GTTACGTGAGCTAAGGGATCAT	At1G07410
4	RabA2B Rev	GAGAGCGACCATCTTCATCAG	At1G07410
5	ABCC4 Fw	GTATCTTACACGCTCCCATGTC	At2G47800
6	ABCC4 Rev	GACCACAAGTCCGAGCATAAA	At2G47800
7	OGHF Fw	CGGTTTACGATGTTGGTCTTCT	At3G55430
8	OGHF Rev	GACCTCGCTACACACCATTTAC	At3G55430
9	SQE3 Fw	CGAGTGCTTCAGGGATCATATT	At4G37760
10	SQE3 Rev	GGAGTAGGAGGAGCTCTGTAAA	At4G37760
11	GDSL_Fw	CCACCTCCCTATCTCTCACTTA	At5G55050
12	GDSL_Rev	TGGTCGGAACTGTTGAAGATT	At5G55050

Supplementary table 2: List of plasmid used for cloning and developing transgenic in this studies.

	Plasmid	Description	Antibiotic Selection		Reference
			E.coli	At	
1	TOPO-D-pENTR	LR cloning entry vector from Thermo-fisher	Kana	NA	Thermo-fisher Cat. No K2400-20
2	pENTR_gene:RabA2b	Entry clone of RabA2b gene for Gateway LR cloning	Kana	NA	This work
3	pENTR_Pro:RabA2b	Entry clone of RabA2b Promoter for Gateway LR cloning	Kana	NA	This work
4	pK7WGF2.0	Destination Gateway vector for translational fusion of RabA2b with N-terminal eGFP	Spect	Kana	(Karimi et al., 2002)
5	pKGWFS7.0	Destination Gateway vector for transcriptional fusion of RabA2b promoter upstream of GUS	Spect	Kana	(Karimi et al., 2002)
6	Pro35S::PIP2A-mCherry (CD3-1008)	Plasma membrane marker expressing mCherry:PIP2A under 35S promoter	Spect	Basta	(Nelson et al., 2007)
7	Pro35S::GFP-RabA2b	RabA2b gene cloning in destination vector pK7WGF2.0 by LR cloning reaction with entry vector pENTR_gene:RabA2b to develop translational fusion vector expressing RabA2b with N-terminal eGFP under 35S promoter.	Spect	Kana	This work
8	ProRabA2b-GUS	RabA2b promoter cloning in destination vector pKGWFS7.0 by LR cloning reaction with entry vector pENTR_promoter:RabA2b to develop transcriptional fusion construct of GUS gene under RabA2b promoter	Spect	Kana	This work

Spect - Spectinomycin (50 µg/mL),
Kana – Kanamycin (50 µg/mL) and
Basta - Glufosinate-ammonium (15 µg/mL)

Table S3. List of GO-terms related to cellular component for proteins identified in the membrane enriched fraction of all the three genotype.

GO ID	Cellular component GO Term	Gene	FDR
GO:0044464	Cell part	3709	1.20E-148
GO:0016020	Membrane	2397	2.20E-307
GO:0044422	Organelle part	1616	8.60E-232
GO:0005886	Plasma membrane	1394	1.50E-233
GO:0032991	Macromolecular complex	851	3.10E-99
GO:0031090	Organelle membrane	690	2.30E-110
GO:0055044	Symplast	524	5.90E-133
GO:0030054	Cell junction	524	1.00E-132
GO:0098805	Whole membrane	454	8.80E-95
GO:0098796	Membrane protein complex	281	1.10E-54
GO:0034357	Photosynthetic membrane	238	1.20E-56

False detection rate (FDR)
